# Interactions between large molecules pose a puzzle for reference quantum mechanical methods

**DOI:** 10.1038/s41467-021-24119-3

**Published:** 2021-06-24

**Authors:** Yasmine S. Al-Hamdani, Péter R. Nagy, Andrea Zen, Dennis Barton, Mihály Kállay, Jan Gerit Brandenburg, Alexandre Tkatchenko

**Affiliations:** 1 Department of Chemistry, University of Zurich, Zürich, Switzerland; 2 Department of Physics and Materials Science, University of Luxembourg, Luxembourg, Luxembourg; 3 Department of Physical Chemistry and Materials Science, Budapest University of Technology and Economics, Budapest, Hungary; 4 Dipartimento di Fisica Ettore Pancini, Università di Napoli Federico II, Monte S. Angelo, Napoli, Italy; 5 Department of Earth Sciences, University College London, London, UK; 6 Thomas Young Centre, University College London, London, UK; 7 London Centre for Nanotechnology, University College London, London, UK; 8 Merck Data Office, Merck KGaA, Darmstadt, Germany

**Keywords:** Chemical physics, Quantum chemistry, Electronic structure

## Abstract

Quantum-mechanical methods are used for understanding molecular interactions throughout the natural sciences. Quantum diffusion Monte Carlo (DMC) and coupled cluster with single, double, and perturbative triple excitations [CCSD(T)] are state-of-the-art trusted wavefunction methods that have been shown to yield accurate interaction energies for small organic molecules. These methods provide valuable reference information for widely-used semi-empirical and machine learning potentials, especially where experimental information is scarce. However, agreement for systems beyond small molecules is a crucial remaining milestone for cementing the benchmark accuracy of these methods. We show that CCSD(T) and DMC interaction energies are not consistent for a set of polarizable supramolecules. Whilst there is agreement for some of the complexes, in a few key systems disagreements of up to 8 kcal mol^−1^ remain. These findings thus indicate that more caution is required when aiming at reproducible non-covalent interactions between extended molecules.

## Abstract (web summary)

Quantum-mechanical methods of benchmark quality are widely used for describing molecular interactions. The present work shows that interaction energies by CCSD(T) and DMC are not in consistent agreement for a set of polarizable supramolecules calling for cooperative efforts solving this conundrum.

## Introduction

The most accurate methods for studying matter at the atomic scale are wavefunction-based approaches, which explicitly account for many-electron interactions. Given only the positions and nuclear charges of atoms, we can now predict, among basically every observable property, the binding strength of relatively small molecular systems (i.e., <50 atoms) to within a few tenths of a kcal mol^−1^ using many-body solutions to the Schrödinger equation^1–3^. This value is better than the so-called “chemical accuracyˮ of 1 kcal mol^−1^ required for reliable predictions of thermodynamic properties. Indeed, the relative stabilities of many non-covalently bound materials such as 2D layered materials, pharmaceutical drugs, and different polymorphs of ice, are underpinned by small energy differences on the order of tenths of a kcal mol^−1^^4^. However, experimentally determining binding affinities under well-defined, pristine conditions is notoriously challenging^5^. In addition, thousands of computational works describe physical interactions in materials, which are not well understood at the experimental level, for instance, as part of rational design initiatives in novel materials including soft colloidal matter, nanostructures, metal organic, and covalent organic frameworks^6–8^. The present shortage of benchmark information is a major setback for forming reliable predictions across the natural sciences and is frequently addressed through demanding, but increasingly feasible, wavefunction-based methods. However, extending the use of highly-accurate methods to a regime of larger molecules is hindered by theoretical and technical challenges due to the steep increase in computational cost required for an accurate description of many-electron interactions^9,10^.

Here we use two widely trusted wavefunction methods that can provide sub-chemically accurate solutions to the electronic Schrödinger equation for non-covalent interactions. First, we utilize coupled-cluster (CC) theory with single, double, and perturbative triple excitations [CCSD(T)]^11^—approximated via the local natural orbital (LNO) scheme to be practicable [LNO-CCSD(T)]^12,13^. Coupled cluster theory has gained great prominence in the last 30 years and the label of ‘gold-standard’ for remarkable accuracy on virtually all systems in its domain of applicability^14^. Second, a stochastic quantum method that computes the energy for the many-electron wavefunction directly is known as fixed-node diffusion Monte Carlo (FN-DMC)^15^. This method has seen a surge of use in recent years, particularly for predicting large molecules and periodic systems with non-covalent interactions^10,16,17^, such as molecular crystals^18,19^ and adsorption on 2D materials^16,20–22^. The accuracy and suitability of FN-DMC in complex non-covalently bound extended materials has been established through excellent agreement with a wealth of different experiments. For example FN-DMC has accurately predicted the binding energy of bilayer graphene^17^ and the cohesive energies of water ice polymorphs^18^, as well as of carbon dioxide, ammonia, benzene, naphthalene, and anthracene crystals^19^. These constitute highly-polarizable materials with significant long-range van der Waals interactions.

**Fig. 1:**
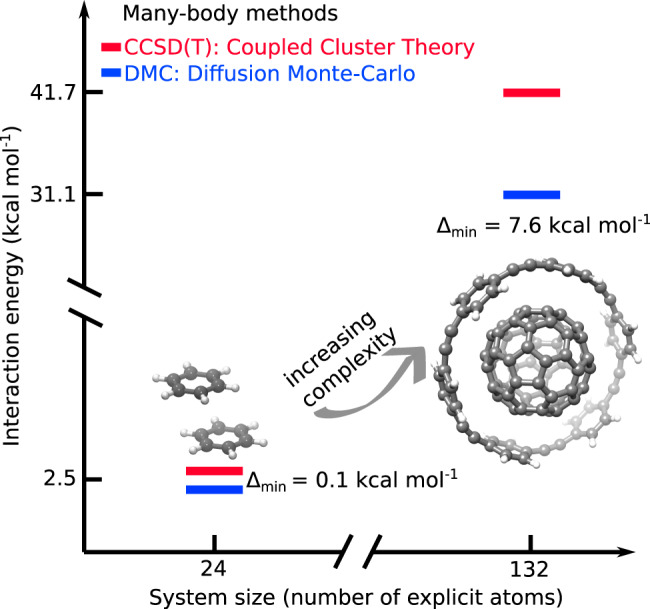
CCSD(T) and FN-DMC interaction energies for a medium and a large dimer. It can be seen that the interaction energy increases by a factor ~10, near-linearly with the size of the system, whereas the corresponding disagreement between CCSD(T) and FN-DMC increases by a factor of ~76.

As we demonstrate in Fig. 1 and Table 1, CCSD(T) and FN-DMC interaction energies are in sub-chemical agreement in small systems such as the benzene-water dimer^21^ and the dimers of benzene, pyridine, and uracil. Nonetheless, FN-DMC and CCSD(T) are still prohibitively expensive for most applications in biology and chemistry, and as result, very little is known about how predictive these theoretical methods are in the regime of medium-to-large polarizable molecules.

**Table 1:** Interaction energies in kcal mol^−1^ for best estimated CCSD(T) and FN-DMC, as well as their minimum differences (${{{\Delta }}}_{\min }$) for dimers taken form the S66 compilaton, for the L7 supramolecular data set and the buckyball-ring complex (C_60_@[6]CPPA). The indicated errors for CCSD(T) are extrapolated from the convergence of basis sets and local approximations in LNO-CCSD(T). The errors indicated in FN-DMC interaction energies account for the stochastic uncertainty of the estimation, and identifies a 95% confidence interval (i.e., ± 2*σ*).

Complex	No. of atoms	CCSD(T)	FN-DMC	${{{\Delta }}}_{\min }$ ^a^
pyridine-pyridine PD	22	−3.70 ± 0.08	−3.51 ± 0.20	0.0
pyridine-pyridine TS	22	−3.48 ± 0.06	−3.44 ± 0.20	0.0
benzene-pyridine PD	23	−3.28 ± 0.07	−3.03 ± 0.16	0.0
benzene-pyridine TS	23	−3.24 ± 0.05	−3.08 ± 0.16	0.0
pyridine-uracil PD	23	−6.61 ± 0.09	−6.38 ± 0.18	0.0
benzene-benzene PD	24	−2.67 ± 0.07	−2.38 ± 0.12	*0.1*
benzene-benzene TS	24	−2.81 ± 0.06	−2.71 ± 0.12	0.0
uracil-uracil PD	24	−9.61 ± 0.10	−9.40 ± 0.16	0.0
benzene-uracil PD	24	−5.48 ± 0.11	−5.11 ± 0.18	*0.1*
GGG	48	−2.1 ± 0.2	−1.5 ± 0.6	0.0
CBH	112	−11.0 ± 0.2	−11.4 ± 0.8	0.0
GCGC	58	−13.6 ± 0.4	−12.4 ± 0.8	*0.1*
C3A	87	−16.5 ± 0.8	−15.0 ± 1.0	0.0
C2C2PD	72	−20.6 ± 0.6	−18.1 ± 0.8	**1.1**
PHE	87	−25.4 ± 0.2	−26.5 ± 1.3	0.0
C3GC	101	−28.7 ± 1.0	−24.2 ± 1.3	**2.2**
C_60_@[6]CPPA	132	−41.7 ± 1.7	−31.1 ± 1.4	**7.6**

^a^ ${{{\Delta }}}_{\min }$ is 0.0 for statistically indistinguishable results. Thermodynamically consistent ${{{\Delta }}}_{\min }$ is highlighted in italics and inconsistent ${{{\Delta }}}_{\min }$ is highlighted in bold.

**Fig. 2:**
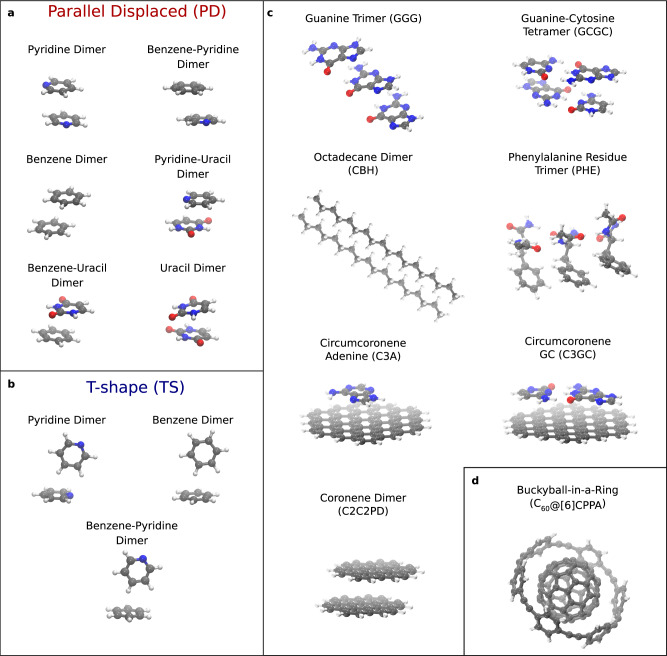
Molecular complexes computed in this work. **a** Parallel displaced (PD) dimers of benzene, pyridine, and uracil molecules from the S66 data set^27^. **b** T-shape (TS) dimers of pyridine and benzene from S66. **c** The supramolecular complexes from L7 data set^28^ and (**d**) a buckyball-ring supramolecular complex consisting of 132 atoms.

Straightforward extrapolations of interactions from small molecules to large complexes are difficult to make due to the interplay and accumulation of interactions that are non-additive, anisotropic, or have many-body character^21,23–26^. As such, a deeper understanding of non-covalent interactions can be gained by directly applying state-of-the-art methods in larger molecular complexes. Here, we use frequently studied molecular data sets: a subset of the S66 by Řezáč et al.^27^ and the full L7 molecular data set from Sedlak et al.^28^ to ascertain the predictive power of FN-DMC and CCSD(T) for medium to relatively large complexes involving intricate *π* − *π* stacking, electrostatic interactions, and hydrogen-bonding (see Fig. 2). In addition, we consider a larger system of a C_60_ buckyball inside a [6]-cycloparaphenyleneacetylene ring (which we label as C_60_@[6]CPPA), consisting of 132 atoms. This structure has a number of interesting features: (i) an open-framework that can be found in covalent organic frameworks and carbon nanotubes, (ii) the buckyball has a large polarizability (76 ± 8 Å^3^)^29^ which gives rise to considerable dispersion interactions, and (iii) confinement between the ring and the buckyball that may cause non-trivial long-range repulsive interactions^30,31^.

Following recent algorithmic advances for more efficient CCSD(T) and FN-DMC, we predict interaction energies for a set of medium-sized polarizable organic dimers and supramolecular complexes, and converge numerical thresholds to the best of our joint knowledge and expertize. Hereafter, we refer to CCSD(T) and FN-DMC interaction energies but note that a number of approximations are used in both methods. More specifically, the CCSD(T) interaction energies we report come from systematically converging LNO-CCSD(T) toward canonical CCSD(T) and accompanied with corresponding uncertainty estimates. Meanwhile, the significance of approximations in FN-DMC interaction energies are assessed using statistical measures where error bars indicate 95% confidence intervals. Furthermore, to define agreement between CCSD(T) and FN-DMC energies, we take into account the uncertainty estimates and a physically relevant energy window that is room temperature k_b_T or 0.6 kcal mol^−1^. First, interaction energies that differ by less than the combined error estimates from CCSD(T) and FN-DMC are statistically indistinguishable. Second, interaction energies which are different by <0.6 kcal mol^−1^ outside of the combined error estimates are thermodynamically consistent. Above a difference of 0.6 kcal mol^−1^ outside of error bars the interaction energies are inconsistent, indicating disagreement between the methods.

In seven out of nine medium-sized highly-polarizable organic molecules computed here, CCSD(T) and FN-DMC interaction energies are indistinguishable and thermodynamically consistent in the remaining two. Similarly, CCSD(T) and FN-DMC interaction energies are either indistinguishable or thermodynamically consistent for five of the eight supramolecular complexes we consider, covering a range of interactions including hydrogen-bonding and *π* − *π* stacking. However, we find that three key complexes reveal several kcal mol^−1^ differences between best estimated CCSD(T) and FN-DMC calculations. Most notably, a substantial disagreement of 7.6 kcal mol^−1^ (or 20%) is found in the interaction energy (*E*_int_ as defined in Methods) of the buckyball-ring system. This 7.6 kcal mol^−1^ inconsistency remains on top of the uncertainty estimates incorporating all controllable sources of errors. We also gauge the impact of approximations intrinsic to each method, not covered in the numerical uncertainty estimates, and find that 7.6 kcal mol^−1^ is an order of magnitude beyond these. It is thus yet unclear whether this discrepancy would also be present between the approximation-free CCSD(T) and DMC results or it is a result of an unexplored source of error. As shown in Fig. 1 and in Table 1 below, such a sizable deviation cannot be explained solely by the size-extensive growth of the difference between CCSD(T) and FN-DMC. Consequently, the interaction energies of three of the supramolecular complexes considered here are still unsettled.

We applied two different, widely-used and well-performing DFT approaches developed for capturing long-range dispersion interactions: DFT + D4^32^ and DFT + MBD^33^. Both methods model London dispersion based on a coarse-grained description and account for all orders of many-body dispersion in different manner. See refs. ^34,35^ for an overview of various ways to capture dispersion in the DFT framework. We find that DFT + MBD closely matches FN-DMC, while the recent DFT+D4 method agrees well with CCSD(T), irrespective of the level of disagreement between CCSD(T) and FN-DMC. Therefore, the absence of either CCSD(T) or FN-DMC references could incorrectly suggest that one of the DFT methods performs better than the other. This illustrates that the unprecedented level of disagreement amongst state-of-the-art methods in large organic molecules has consequences well outside the developer communities.

CCSD(T) and FN-DMC methods account for dynamic electron correlation through an expansion in electron configurations in the former and through the projection to the ground state wave function in the latter. These two equally viable formulations can be illustrated by the corresponding expressions of Ψ(**R**), the exact wavefunction:

1. **DMC:** A propagation according to the imaginary time Schrödinger equation is performed to project out the ‘exact’ electronic ground state from a trial function Ψ_T_(**R**):
  1 $$\left|{{\Psi }}({\bf{R}})\right\rangle =\mathop{\lim}\limits_{\tau \to \infty }\exp \ \left[-\tau (\hat{H}-{E}_{{\rm{T}}})\right]\left|{{{\Psi }}}_{{\rm{T}}}({\bf{R}})\right\rangle$$
2. **CC:** Expansion of excited determinants generated via the operator ${\hat{T}}_{n}$ from a reference wavefunction:
  2 $$\left|{{\Psi }}({\bf{R}})\right\rangle =\exp \ \left[\mathop{\sum }\limits_{n=1}^{N}{\hat{T}}_{n}\right]\left|{{{\Psi }}}_{{\rm{T}}}({\bf{R}})\right\rangle$$

The crucial challenge lies in extensively accounting for relatively small fluctuations in the electron charge densities. To this end, DMC is a stochastic approach, where the wavefunction is described through a set of configurations, otherwise referred to as walkers. Walkers evolve in imaginary time through discrete steps of size Δ*τ*. The stochastic uncertainty associated with any DMC evaluation is inversely proportional to the square root of the sampling. In order to make this propagation efficient for an electronic wavefunction a few approximations are typically employed: the fixed node (FN) constraint^15^, the use of pseudopotentials^36–39^, and solutions enhancing the stability of walker populations^40,41^. In non-covalent interactions, the challenge for FN-DMC is to provide precise and accurate evaluations of the interaction energy *E*_int_, despite *E*_int_ being a tiny fraction of the total energy, e.g., it is circa 1/10^4^ of the total energy in the C_60_@[6]CPPA complex. Precision is achieved by exploiting the almost perfect scaling of DMC on modern supercomputer facilities^42–44^ and thanks to recent algorithmic improvements which reduced the time-step bias and made DMC up to 100 times more efficient^40^. The FN-DMC setup here employed has been used and validated against experiments and CCSD(T) a number of times, for instance in refs. ^18,19,21,45^.

**Fig. 3:**
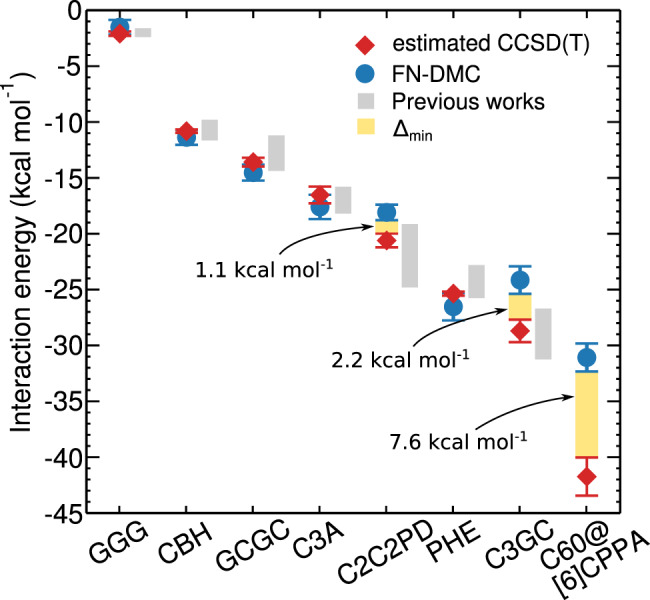
CCSD(T) and FN-DMC interaction energies for supramolecular complexes. The L7 data set^28^ and the C_60_@[6]CPPA buckyball-ring complex are arranged in terms of increasing interaction strength. Gray bars mark the range of interaction energies reported in the literature using alternative wavefunction-based methods (e.g., QCISD(T)^28^, and various local CCSD(T) approaches^49,51,52,67–70^). The yellow bars indicate the delta value (${{{\Delta }}}_{\min }$) which is the minimum difference between best converged CCSD(T) and FN-DMC, given by the estimated and stochastic error bars (with 95% confidence interval), respectively.

In coupled cluster theory, non-covalent interactions require a high-order treatment of many-electron processes, as is included in CCSD(T), and a sufficiently large single-particle basis set. Reaching basis set saturation and well-controlled local approximations concurrently for the studied systems required previously unfeasible computational efforts as shown by the several kcal mol^−1^ scatter of interaction energy predictions reported for the L7 set (see Fig. 3). Our recent efforts enabled the following: (i) a systematically converging series of local CCSD(T) results is presented for highly-complicated complexes, (ii) both the local and the basis set incompleteness (BSI) errors are closely monitored using comprehensive uncertainty measures^13^, (iii) convergence up to chemical accuracy is reached for the complete L7 set concurrently in the local approximations as well as in the basis set saturation.

The benefit of such demanding FN-DMC and CCSD(T) convergence studies is that the resulting interaction energies, up to the respective error bars, can be considered independent of the corresponding approximations. Consequently, we expect that the CBS limit of the exact CCSD(T) results could, in principle, be approached similarly using alternative basis sets^10,46,47^ or local correlation methods^48–51^. For instance, using different basis set corrections and local approximations for CCSD(T), but an error estimate reminiscent of our approach presented here, the most recent L7 interaction energies of ref. ^52^ are identical to ours within the corresponding error estimates. In addition, FN-DMC interaction energies for the L7 complexes have been reported very recently that are in close agreement with our results, even though a different algorithm and implementation has been employed^53^.

We use highly-optimized algorithms both for FN-DMC and CCSD(T) as outlined in Methods, and push them beyond the typically applied limits. We used circa 0.7 and 1 million CPU core hours for FN-DMC and CCSD(T), respectively. This is equivalent to running a modern 28 core machine constantly for ~7 years.

## Results

### Consensus in medium-sized highly-polarizable organic molecules

Demonstrating agreement between fundamentally different electronic structure methods for solving the Schrödinger equation provides a proof-of-principle for the accuracy of the methods beyond technical challenges. To date, disagreements beyond 1 kcal mol^−1^ on molecular systems between CCSD(T) and FN-DMC have been reported for systems where key approximations, e.g., single-reference wavefunction, accurate node-structure, and basis set completeness, were not completely fulfilled^54–56^. Previously however, CCSD(T) and FN-DMC were found in agreement within the error bars, for the interaction energies of small organic molecules with pure dynamic correlation^9,16,57^ as well as some extended systems^19,21,58^.

Here we extend the list of systems with consistent CCSD(T) and FN-DMC interaction energies with nine medium-sized dimers of 22–24 atoms taken from the S66 compilation^27^. Interaction energies and corresponding errors estimates are collected in Table 1 for the parallel displaced (PD) and T-shaped (TS) dimers of benzene, pyridine, and uracil (see Fig. 2). The level of uncertainty in our results throughout this paper is indicated by the sum of local and BSI error estimates for CCSD(T). In FN-DMC the main source of uncertainty is the stochastic nature of the approach, which is here accounted for by reporting a confidence interval of 95% (i.e., ± two standard deviations). To err on the side of finding agreement, we define ${{{\Delta }}}_{\min }$ as the absolute minimum difference between best converged CCSD(T) and FN-DMC for the closest limits of the corresponding error bars, i.e., the smallest deviation between the methods according to the uncertainty estimates.

For the majority of medium-sized complexes in Table 1 FN-DMC and CCSD(T) interaction energies are statistically indistinguishable as ${{{\Delta }}}_{\min }=0$ kcal mol^−1^, with the exception of the benzene-uracil and the benzene PD dimers.

The benzene PD dimer has garnered much interest as a prototypical example of *π* − *π* stacking interaction. Previous predictions of the interaction energy from CCSD(T) are −2.69 kcal mol^−1^^59^ in excellent agreement with our LNO-CCSD(T) result of −2.67 ± 0.07 kcal mol^−1^ (see Table 1). However, previous FN-DMC predictions of the benzene PD dimer use marginally different structures and algorithms, resulting in a wide range of predicted interaction energies^60–62^. Here, using the latest DMC algorithms and well-converged stochastic error bars we predict −2.38 ± 0.12 kcal mol^−1^ to be interaction energy of the PD benzene dimer (S66 structure) from FN-DMC. This result is robust with respect to different nodal structures (as can be seen in Supplementary Note 2 B) and is therefore unlikely to be affected by the fixed-node approximation. This leaves a −0.29 ± 0.19 kcal mol^−1^ discrepancy, or ${{{\Delta }}}_{\min }=0.1$ kcal mol^−1^ between FN-DMC and CCSD(T), which is 11 ± 7% of the interaction energy. While the relative discrepancy can be considered non-negligible, evidently the absolute energy difference is well within thermodynamic consistency. Therefore, even with well-defined error bars, CCSD(T) and FN-DMC interaction energies are thermodynamically consistent for weakly interacting medium-sized dimers.

### Losing consensus on supramolecular interactions

Establishing agreement for systems at the 100 atom range has been hindered by the sizable or unavailable error estimates for finite systems^9^. For example, binding energies of large host-guest complexes derived from experimental association free energies^63,64^ motivated previous FN-DMC^65^ as well as local CCSD(T)^66^ computations. While the average discrepancy of these FN-DMC and local CCSD(T) binding energies was found to be about 2.4 kcal mol^−1^, it is not possible to make conclusive remarks on the consistency of these results. Uncertainty estimates are unavailable for local CCSD(T), but could be comparable to the average discrepancy, while the error estimates reported for both experimental and FN-DMC energies reach up to a few kcal mol^−1^.

Here, we consider similar but somewhat smaller supramolecular complexes (Fig. 2) and obtain tightly converged local CCSD(T) and FN-DMC results sufficient for rigorous comparisons (see Fig. 3 and Table 1). The complexes are arranged in Fig. 3 according to increasing interaction strength, which roughly scales with the size of the interacting surface. CCSD(T) and FN-DMC agree on the interaction energy to within 0.1 kcal mol^−1^, taking error bars into account, for a subset of the complexes we consider: GGG, CBH, GCGC, C3A, and PHE. These complexes are between 48 and 112 atoms in size and exhibit *π* − *π* stacking, hydrogen-bonding, and dispersion interactions. Therefore, the agreement for these five complexes indicates their absolute interaction energies are established references and can be used to benchmark other methods for large molecules. Here, relative differences of very small interaction energies have to be interpreted carefully as they are sensitive to the uncertainty estimates. In GGG for example, the results are statistically indistinguishable whilst the relative disagreement is up to 65%. In contrast, the relative disagreement between FN-DMC and CCSD(T) is better resolved in the more strongly interacting C_60_@[6]CPPA complex, at 18–33%.

A salient and surprising finding is the disagreement between state-of-the-art methods on the interaction energy of three non-trivial complexes: coronene dimer (C2C2PD), circumcoronene-GC base pair (C3GC), and buckyball-ring (C_60_@[6]CPPA). The minimum differences (${{{\Delta }}}_{\min }$), as indicated in Table 1 and Fig. 3 are 1.1, 2.2, and 7.6 kcal mol^−1^ for C2C2PD, C3GC, and C_60_@[6]CPPA, respectively, but the disagreements could be as high as 3.9, 6.9, and 13.7 kcal mol^−1^, respectively. Considering the comparable size of C3A, PHE, and CBH to C2C2PD, C3GC, and C_60_@[6]CPPA, the ${{{\Delta }}}_{\min }$ values of the latter three complexes are not explained simply by the large size or the large area of the interacting surface. CCSD(T) predicts consistently stronger interaction in these complexes than FN-DMC, but at this point it is unclear what the exact interaction energies are.

C2C2PD has attracted the most attention to date in the CCSD(T) context as it represents a stepping stone between two widely studied systems: benzene dimer and graphene bilayer^9^. Already C2C2PD has posed a significant challenge to various local CCSD(T) methods due to its slowly-decaying long-range interactions^13,49,51,52,67–70^. Considerable efforts have been devoted recently^13,49,51^ to narrow down the local CCSD(T) interaction energy of C2C2PD to the range of about −19 to −21 kcal mol^−1^. Thus the presently reported −20.6 ± 0.6 kcal mol^−1^ interaction energy and previous local CCSD(T) results, containing analogous local approximations, consistently indicate stronger interaction than FN-DMC for C2C2PD. This trend, to a smaller extent, is also seen in the PD benzene complex and size-extensive error propagation might be expected, but it is clearly insufficient to explain 18–31% relative disagreement found in C_60_@[6]CPPA for example.

### Distinct errors using DNA base molecules on circumcoronene

The C3GC and C3A complexes are ideal for assessing the convergence of CCSD(T) and FN-DMC, due to their chemical similarity and importance of *π* − *π* stacking interactions, i.e., nucleobases stacked on circumcoronene. CCSD(T) and FN-DMC agree within 1 kcal mol^−1^ for the interaction energy of C3A, whereas there is a notable disagreement of at least 2.2 kcal mol^−1^ in the interaction energy of C3GC. Interestingly, both systems involve similar interaction mechanisms, with C3GC exhibiting both stacking and hydrogen-bonding interactions.

**Fig. 4:**
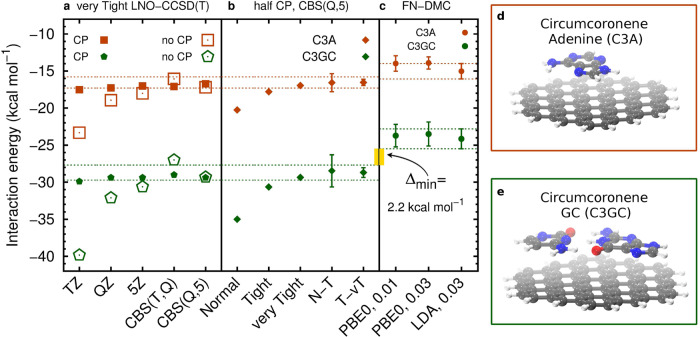
The interaction energy of the C3A and C3GC complexes using different settings. LNO-CCSD(T) shown in (**a** and **b**), and FN-DMC in (**c**). The orange and green dashed horizontal lines, for C3A and C3GC, respectively, enclose the best estimated CCSD(T) (**a** and **b**) and the final FN-DMC (**c**) interaction energies using the corresponding uncertainty estimates and stochastic error bars. The FN-DMC error bars indicate 95% confidence intervals. The yellow bar denotes the minimum difference between CCSD(T) and FN-DMC (${{{\Delta }}}_{\min }$). **a** CP-corrected and uncorrected LNO-CCSD(T) interaction energies using the aug-cc-pV*X*Z basis sets, as well as CBS(*X*,*X* + 1) extrapolation. **b** Convergence of half CP-corrected LNO-CCSD(T)/CBS(Q,5) interaction energies using a series of LNO thresholds as well as Normal--Tight (N–T) and Tight--very Tight (T-vT) extrapolations. **c** FN-DMC interaction energies with two nodal surfaces or C3GC from DFT (PBE0 and LDA) and different time-steps (given in a.u.) for C3A and C3GC. **d** C3A complex. **e** C3GC complex.

CCSD(T) and FN-DMC interaction energies involve multiple approximations. In Fig. 4 we analyze the most critical approximations for each method on the example of the C3A and C3GC complexes, and we also consider the other remaining known sources of error in Methods.

In obtaining CCSD(T) interaction energies, the sources of error are:

- Single-particle basis representation of the CCSD(T) wavefunction.
- Local approximations of long-range electron correlation according to the LNO scheme.
- Neglected core electron correlation.
- Missing high-order many-electron contributions beyond CCSD(T).

For the single-particle basis representation in CCSD(T) we employed conventional correlation-consistent basis sets augmented with diffuse functions^71^, aug-cc-pV*X*Z (*X* = T, Q, and 5) as shown in panel a) of Fig. 4. The remaining BSI is alleviated using extrapolation^72^ toward the complete basis set (CBS) limit [CBS(*X*,*X* + 1), *X* = T, Q], and counterpoise (CP) corrections^73^. The local errors decrease systematically as the LNO threshold sets are tightened (Normal, Tight, very Tight) enabling extrapolations, e.g., Normal–Tight (N–T), to estimate the canonical CCSD(T) interaction energy^13^ (see panel b) of Fig. 4). Exploiting the systematic convergence properties, an upper bound for both the local and the BSI errors can be given without relying on their potential cancellation of errors (see Methods).

Benchmarks presented previously for energy differences of a broad variety of systems showed excellent overall accuracy at the Normal–Tight extrapolated LNO-CCSD(T)/CBS(T,Q) level (M1)^13^. However, the BSI error bar of 1.0 kcal mol^−1^ and the local error bar of 2.2 kcal  mol^−1^ obtained for C3GC at this M1 level are impractical for a definitive comparison with FN-DMC. The next steps along both series of approximations toward chemical accuracy, i.e., the use of very Tight LNO thresholds and the aug-cc-pV5Z basis set (M2), have been enabled by our recent method development efforts^12,13,74^. With these better converged interaction energies, the M2 level uncertainty estimates are up to a factor of three smaller than at the M1 level. Explicitly, 0.7 (0.4) kcal mol^−1^ local (BSI) error estimate is obtained for C3GC. The same measures are the largest for C_60_@[6]CPPA at the M2 level being 1.1 and 0.6 kcal mol^−1^, respectively. Moreover, for the remaining L7 complexes, the local (BSI) uncertainty estimates indicate even better convergence of 0.1–0.4 (0.1–0.3) kcal mol^−1^. Additional details are provided in Methods and in Supplementary Note 1 of the Supplementary Material (SM).

Known sources of error to consider in our FN-DMC calculations are:

- The fixed-node approximation which restricts the nodal-structure to that of the input guiding wavefunction.
- Time-step bias from the discretization of imaginary time for propagating the wavefunction.
- Pseudopotentials to approximate core electrons for each atom.

First, we analyze the most pertinent source of error in FN-DMC which is the fixed-node approximation. The different nodal surfaces from DFT methods serve to indicate the dependence of the FN-DMC interaction energy on the nodal structure. Indeed, from Fig. 4, we find no indication that the FN-DMC interaction energies of C3GC is affected by the nodal structure with the results being statistically indistinguishable. This shows that DMC estimations are robust for different ways to initialize the orbitals in a Slater-Jastrow ansatz. However, we cannot conclude that the FN bias is negligible, as it is possible that a more involved multireference ansatz yields different results. Unfortunately, a multireference ansatz implies a much larger computational effort, which is not yet possible on the large systems discussed here. Second, FN-DMC energies are sensitive to the time-step and we rely on recent improvements in FN-DMC algorithms^36,40^, that enable convergence of time-steps as large as 0.05 a.u. We used 0.03 a.u. and 0.01 a.u. time-steps to compute the interaction energies of C3A and C3GC. Figure 4 indicates that the interaction energy is statistically indistinguishable for the different time-steps considered here for both C3A and C3GC. The time-step and fixed-node approximations perform similarly well for the coronene dimer and the buckyball-ring complex (see Supplementary Note 2 C and D of the SM). Third, recently reported all-electron FN-DMC interaction energies for the L7 complexes^53^ are in agreement with our pseudopotential-based FN-DMC results. Therefore, our FN-DMC interaction energies are also robust with respect to the use of pseudopotentials.

### Open challenges for next generation of many-body methods

CCSD(T) and FN-DMC have been shown to agree with sub-chemical accuracy for small organic dimers^9,16,57^, molecular crystals^18,19^, and small physisorbed molecules on surfaces^21,58^. Indeed, we also find good agreement in the absolute interaction energies for five of the eight complexes considered here. However, we find that the disagreement by several kcal mol^−1^ in C_60_@[6]CPPA particularly, cannot be explained by the controllable sources of error. While both methods are highly sophisticated, they are still approximations to the exact solution of the many-electron Schrödinger equation. Moreover, there can be non-trivial coupling between approximations within each method, which remain poorly understood for complex many-electron wavefunctions.

#### Are we there yet with FN-DMC?

The reported interaction energies of C2C2PD, C3GC, and C_60_@[6]CPPA indicate that FN-DMC stabilizes the interacting complexes more weakly than estimated CCSD(T). Therefore, one possibility for the discrepancy between the methods is that FN-DMC (as applied here) does not capture the correlation energy in the bound complexes sufficiently. Reasons for this can include the fixed-node approximation and more generally, insufficient flexibility in the wavefunction ansatz.

The Slater-Jastrow ansatz was applied here using a single determinant combined with a Jastrow factor containing explicit parameterizable functions to describe electron-electron, electron-nucleus, and electron-electron-nucleus interactions. We have evaluated FN-DMC interaction energies for different nodal structures for C3GC, C2C2PD, C_60_@[6]CPPA and in all cases the FN-DMC interaction energies are in 1-*σ* agreement (see Supplementary Note 2) with stochastic errors that are mostly under 1 kcal mol^−1^. Among these systems, the largest potential deviation (Δ_max_) due to the fixed-node error is estimated to be ~ 3.7 kcal mol^−1^ in C_60_@[6]CPPA. Although this potentially large source of error is not enough to explain the 7.6 kcal mol^−1^${{{\Delta }}}_{\min }$ disagreement with CCSD(T), it remains a pertinent issue for establishing chemical accuracy. Reducing the fixed-node error, for example by using more than one Slater determinant to systematically improve the nodal structure, in such large molecules remains challenging^75,76^. Promising alternatives include the Jastrow antisymmetrized geminal power approach which has recently been shown to recover near-exact results for a small, strongly correlated cluster of hydrogen atoms^77^.

The Jastrow factor is a convenient approach to increase the efficiency of FN-DMC since in the zero time-step limit and with sufficient sampling, the FN-DMC energy is independent of this term. However, the quality of the Jastrow factor can be non-uniform for the bound complex and the noninteracting fragments, which can introduce a bias at larger time-steps. The recent DLA method in FN-DMC reduces this effect^36^ and was applied to the C_60_@[6]CPPA complex reported in Table 1 and also tested for GGG, C3A, and C2C2PD complexes (see Methods for further details). In all cases, FN-DMC with DLA is in agreement (95% confidence interval) with non-DLA FN-DMC interaction energies. For example, the C2C2PD FN-DMC interaction energy with DLA is − 17.4 ± 1.0 kcal mol^−1^ whilst with standard LA, it is − 18.1 ± 0.8 kcal mol^−1^. Moreover, the interaction strengths tend toward being weaker with DLA in the systems we consider, i.e., further from the CCSD(T) interaction energies. As such, the discrepancy between FN-DMC and CCSD(T) remains regardless of any potential error from the Jastrow factor in our findings.

We estimate the error from the use of Trail and Needs pseudopotentials^78,79^ in FN-DMC at the Hartree-Fock (HF) level using interaction energy of C2C2PD. We find 0.1 kcal mol^−1^ difference in the HF interaction energy with the employed pseudopotentials and without (i.e., all-electron) which is well within the acceptable uncertainty for our findings. In addition, recently computed all-electron FN-DMC interaction energies of the L7 data set are in agreement with our predictions^53^.

In principle, a more flexible wavefunction ansatz allows a more accurate many-body wavefunction to be reached in DMC, thus recovering electron correlation more effectively. To this end, recently introduced machine learning approaches^80,81^ are promising but more expensive due to the considerable increase in parameters. However, once feasible, a systematic assessment of the amount of electron correlation recovered by these different ansatze in non-covalently bound systems will bring valuable insight to the current puzzle.

#### Potential avenues for improvement upon CCSD(T)

Considering the complexes exhibiting significant *π*-*π* interactions, CCSD(T) is found to predict stronger interaction than FN-DMC. Approximations used for some of the small, long-range energy contributions in local CC methods^49,82^ could potentially lead to overestimated interactions. In the case of the LNO scheme, the majority of the local approximations have marginal effect on the interaction energies when very Tight settings are employed^13^. For the most complicated case of C_60_@[6]CPPA, only 7% (−2.9 kcal mol^−1^) of its interaction energy results from long-range contributions approximated at the second-order, the full many-body treatment up to CCSD(T) level is utilized for the remaining 93% (see Eq. 1 of the SM). While the corresponding 1.1 kcal mol^−1^ error bar appears to estimate the local approximations well (see Supplementary Note 1 B), remaining uncertainties outside of the presented error bars cannot be ruled out.

The employed single-particle basis sets perform exceptionally well for CCSD(T) computations of small molecules^71,72^, but approaching the CBS limit of CCSD(T) for large systems is mostly an uncharted territory in the literature^13,49^. The agreement of CP corrected CBS(T,Q), CBS(Q,5), and uncorrected CBS(Q,5) within 0.06–0.36 kcal mol^−1^ is highly satisfactory (see Supplementary Note 1 A). Currently, Gassian functions based implementations appear to approach the CBS limit of CCSD(T) for extended systems. However, alternative CC methods utilizing plane-wave or real-space representations^10,46,47^ as well as explicitly correlated wavefunction forms^49,51^ could offer advantages to overcome the basis set superposition error and relatively slow convergence associated with Gaussian basis sets for delocalized systems.

The higher-order contribution of three-, four-, etc. electron processes on top of CCSD(T)^83,84^ are usually found to be negligible for weakly-correlated molecules^57^. However, the available numerical experience is limited to complexes below about a dozen atoms, and for some highly-polarizable systems the beyond CCSD(T) treatment of three-electron processes has been shown to contribute significantly to three-body dispersion^85^. The weakly-correlated nature of all complexes is indicated by the perturbative (T) contribution to the total correlation energy component of the CCSD(T) interaction energy being consistently 18–20%. In addition, the CC amplitude based measures all point to pure dynamic correlation (see Supplementary Note 1 B). Due to the extreme computational cost of such higher-order CC computations, it remains an open and considerable challenge to establish whether the contribution of higher-order processes is within sub-chemical accuracy for larger and more complex molecules.

#### Insights from experiments and comparison with density-functional approximations

Experimental binding energies or association constants of supramolecular complexes are particularly valuable, when available, but also have their limitations as back-corrections are needed to separate the effects of thermal fluctuations and solvent effects for example^86^. In the case of C_60_@[6]CPPA for example, the association constant is measured in a benzene solution and indicates a stable encapsulated complex, but one which could not be well-characterized by X-ray crystallography; purportedly due to the rapid rotation of the buckyball guest^87^. Instead, a non-fully encapsulated structure was successfully characterized using toluene anchors on the buckyball. This demonstrates a number of physical leaps that exist between what can be measured and what can be accurately computed.

Other high-level methods, such as the full configuration interaction quantum Monte-Carlo (FCI-QMC) method^10,46^, can be key to assessing the shortcomings from major approximations such as the FN approximation and static correlation. Once the severe scaling with system size associated with FCI-QMC and similar methods is addressed, larger molecules will become feasible. However, in the present time the lack of references in large systems remains a salient problem.

**Fig. 5:**
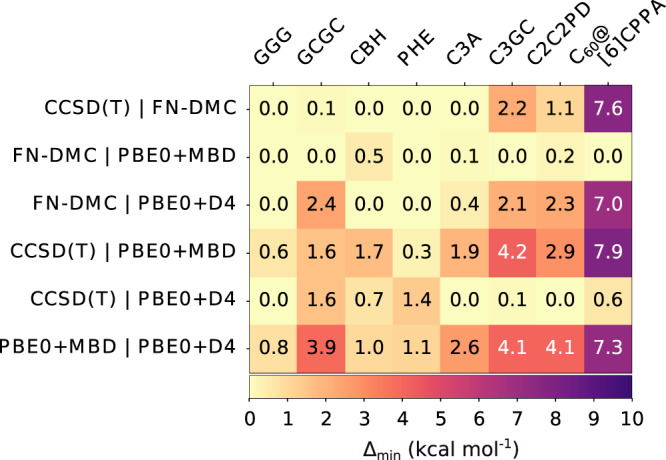
${{{\Delta }}}_{\min }$ is shown between pairs of methods. ${{{\Delta }}}_{\min }$ takes into account the error estimates for CCSD(T) and FN-DMC to show smallest differences with respect to these reference methods. The DFT methods have no quantified uncertainty estimates associated with them. The compared methods are: CCSD(T), FN-DMC, PBE0+MBD and PBE0+D4. The supramolecular complexes are those in the L7 data set and the C_60_@[6]CPPA buckyball-ring complex.

The scarcity of reference information has an impact on all other modelling methods, including density-functional approximations (DFAs), semi-empirical, force field or machine learning based models, etc. which are validated or parameterized based on higher-level benchmarks. In particular, there is a race to simulate larger, more anisotropic, and complex materials, accompanied by a difficulty of choice for modelling methods. To demonstrate the consequences of inconsistent references, Fig. 5 shows interaction energy discrepancies obtained with DFAs, PBE0+D4^32^ and PBE0+MBD^33^, that are both designed to capture all orders of many-body dispersion interactions in different manner. Intriguingly, the PBE0+D4 method is in close agreement with CCSD(T) (mean absolute deviation, MAD = 1.1 kcal mol^−1^), whereas PBE0+MBD is closer to FN-DMC (MAD = 1.5 kcal mol^−1^), but their performance is hard to characterize when CCSD(T) and FN-DMC disagree. Moreover, we decomposed the interaction energies from the DFAs into dispersion components and find that, for C_60_@[6]CPPA the main difference between PBE0+MBD and PBE0+D4 is 6.5 kcal mol^−1^ in the two-body dispersion contribution. Differences in beyond two-body dispersion interactions are smaller and at most 1.6 kcal mol^−1^ in C_60_@[6]CPPA.

## Discussion

Until now, disagreements between reference interaction energies of extended organic complexes have typically been ascribed to unconverged results due to practical bottlenecks. Here, we report highly-converged results at the frontier of wavefunction-based methods; uncovering a disconcerting level of disagreement in the interaction energy for three supramolecular complexes. We have computed interaction energies from CCSD(T) and FN-DMC for a set of supramolecular complexes of up to 132 atoms exhibiting challenging intermolecular interactions. The accuracy of these methods have been repeatedly corroborated in the domain of dozen-atom systems with single-reference character and here we find CCSD(T) and FN-DMC are in excellent agreement for five of the supramolecular complexes suggesting that these methods are able to maintain remarkable accuracy in some larger molecules. However, FN-DMC and CCSD(T) interaction energies disagree by 1.1 kcal mol^−1^ in the coronene dimer (C2C2PD), 2.2 kcal mol^−1^ in GC base pair on circumcoronene (C3GC) and 7.6 kcal mol^−1^ in a buckyball-ring complex (C_60_@[6]CPPA). These disagreements are cemented by reporting sub-kcal mol^−1^ standard deviations in FN-DMC and a systematically converging series of local CCSD(T) interaction energies accompanied by uncertainty estimates approaching chemical accuracy. Therefore, despite our best efforts to suppress all controllable sources of error, the marked disagreement of FN-DMC and CCSD(T) prevents us from providing conclusive reference interaction energies for these three complexes. Such large differences in interaction energies surpass the widely-sought 1 kcal mol^−1^ chemical accuracy and indicate that the highest level of caution is required even for our most advanced tools when employed at the hundred-atom scale.

The supramolecular complexes we report feature *π* − *π* stacking, hydrogen-bonding, and intermolecular confinement, that are ubiquitous across natural and synthetic materials. Thus our immediate goals are to elucidate the sources of the underlying discrepancies and to explore the scope of systems where such deviations between reference wavefunction methods occur. Well-defined reference interaction energies and the better characterization of their predictive power have growing importance as they are frequently applied in chemistry, material, and biosciences. Our findings should motivate cooperative efforts between experts of computational and experimental methods in obtaining well-defined interaction energies and thereby extending the predictive power of first principles approaches across the board.

## Methods

The L7 structures have been defined by Sedlak et al.^28^ and structures can be found on the begdb database^88^. Note that the interaction energy, *E*_int_, is defined with respect to two fragments even where the complex consists of more than two molecules (as in GGG, GCGC, PHE, and C3GC):

3 $${E}_{\text{int}}={E}_{\mathrm{com}}-{E}_{\text{frag}}^{1}-{E}_{\text{frag}\,}^{2}$$

where *E*_com_ is the total energy of the full complex, and ${E}_{\,\text{frag}\,}^{1}$ and ${E}_{\,\text{frag}\,}^{2}$ are the total energies of isolated fragments 1 and 2, respectively. The fragment molecules have the same geometry as in the full complex, i.e., not relaxed. Further details on the configurations can be found in the SM and in ref. ^28^.

The C_60_@[6]CPPA complex is based on similar complexes in previous theoretical and experimental works^65,89,90^ and has been chosen to represent confined *π*-*π* interaction that are numerically still tractable by our methodologies. Its geometry has been symmetrized to *D*_3*d*_ point group, the individual fragments of C_60_ and [6]CPPA are kept frozen (*I*_*h*_ and *D*_6*h*_, respectively). The structure is provided in the SM.

### The local natural orbital CCSD(T) method

In order to reduce the *N*^7^-scaling of canonical CCSD(T) with respect to the system size (*N*), the inverse sixth power decay of pairwise interactions can be exploited (local approximations) and the wavefunction can be compressed further via natural orbital (NO) techniques.^82^ Building on such cost-reduction techniques a number of highly-efficient local CCSD(T) methods emerged in the past decade^12,13,48–51,82,91,92^. As the local approximation-free CCSD(T) energy can be approached by the simultaneous improvement of all local truncations in most of these techniques, in principle, all local CCSD(T) methods are expected to converge to the same interaction energy. Here we employ the local natural orbital CCSD(T) [LNO-CCSD(T)] scheme^12,93^, which, for the studied systems, brings the feasibility of exceedingly well-converged CCSD(T) calculations in-line with FN-DMC. The approximations of the LNO scheme automatically adapt to the complexity of the underlying wavefunction and enable systematic convergence toward the exact CCSD(T) correlation energy, with up to 99.99% accuracy using sufficiently tight settings^13^.

The price of improvable accuracy is that the computational requirements can drastically increase depending on the nature of the wavefunction: while LNO-CCSD(T) has been successfully employed for macromolecules, such as small proteins at the 1000 atom range^12,13^, sizable long-range interactions appearing in the here studied complexes pose a challenge for any local CCSD(T) method^13,48,49,51^. This motivated the implementation of several recent developments in our algorithm and computer code over the lifetime of this project, which cumulatively resulted in about 2–3 orders of magnitude decrease in the time-to-solution and data storage requirement of LNO-CCSD(T)^12,13,93^, and made well-converged computations feasible for all complexes. For instance, we have designed a massively parallel conventional CCSD(T) code specifically for applications within the LNO scheme^94^ and integrated it with our highly optimized LNO-CCSD(T) algorithms^12,13,93^. Here, we report the first large-scale LNO-CCSD(T) applications which exploit the resulted high performance capabilities using the most recent implementation of the Mrcc package^74^ (release date February 22, 2020).

### Computational details for CCSD(T)

The LNO-CCSD(T)-based CCSD(T)/CBS estimates were obtained as the average of CP-corrected and uncorrected (“half CP”)^73^, Tight–very Tight extrapolated LNO-CCSD(T)/CBS(Q,5) interaction energies^13^. Except for C3A, C3GC, and C_60_@[6]CPPA, the CBS(Q,5) notation refers to CBS extrapolation^72^ using aug-cc-pV*X*Z basis sets^71^ with *X* = Q and 5. For C3A, C3GC, and C_60_@[6]CPPA, a Normal LNO-CCSD(T)/CBS(Q,5)-based BSI correction (Δ_BSI_) was added to the Tight–very Tight extrapolated LNO-CCSD(T)/aug-cc-pVTZ interaction energies, exploiting the parallel convergence of the LNO-CCSD(T) energies for these basis sets^13^. Error bars accompanying the LNO-CCSD(T) interaction energies of Fig. 3 and Table 1, and determining the interval enclosed by the dashed lines on panels (a) and (b) of Fig. 4 are the sums of the BSI and local error estimates. The BSI error measure is the maximum of two separate error estimates: the difference between CP-corrected and uncorrected CBS(Q,5) energies, and the difference between CP-CBS(T,Q) and CP-CBS(Q,5) results. This BSI error bar is increased with an additional term if Δ_BSI_ is employed according to Supplementary Note 1 A. Local error bars shown, e.g., on panel (b) of Fig. 4 are obtained via the extrapolation scheme of ref. ^13^. Explicitly, the local error bar of the best estimated CCSD(T) results (see Table S I) is calculated from the difference of the Tight and very Tight LNO-CCSD(T) results evaluated with the largest possible basis sets^13^.

### Computational details for FN-DMC

Our FN-DMC calculations use the Slater-Jastrow ansatz with the single Slater determinants obtained from DFT. The Jastrow factor for each system contains explicit electron-electron, electron-nucleus, and three-body electron-electron-nucleus terms. The parameters of the Jastrow factor were optimized for each complex using the variational Monte Carlo (VMC) method and the varmin algorithm which allows for systematic improvement of the trial wavefunction, as implemented in CASINO v2.13.610^95^. Note that bound complexes were used in the VMC optimizations and the resulting Jastrow factor was used to compute the corresponding fragments. All systems were treated in real-space as non-periodic open systems in VMC and FN-DMC.

We performed FN-DMC simulations using the size-consistent ZSGMA algorithm^40^. Trail and Needs pseudopotentials^78,79^ were used for all elements with the locality approximation (LA) for the non-local pseudopotentials^39^ and 0.03 a.u. time-step for all L7 complexes. Smaller time-steps of 0.003 and 0.01 a.u. were also used to compute the interaction energy of the C2C2PD complex and the interaction energy was found to be in agreement within the stochastic error bars with all three time-steps.

The C_60_@[6]CPPA complex exhibited numerical instability using the standard LA. This prevented sufficient statistical sampling and therefore we computed this complex with two alternative and more numerically stable approaches. First, the energy reported in Fig. 3 and Table 1 is using the recently developed determinant localization approximation (DLA)^36^ implementation CASINO v2.13.809^95^. The DLA gives: (i) better numerical stability than the LA algorithm allowing for more statistics to be accumulated, (ii) smaller dependence on the Jastrow factor, and (iii) addresses an indirect issue related to the use of non-local pseudopotentials. Second, the T-move approximation^38^ (without DLA) was was also applied to C_60_@[6]CPPA for comparison. The T-move scheme is more numerically stable than the standard LA algorithm but is also more time-step dependent and therefore we used results from 0.01 and 0.02 a.u. time-steps to extrapolate the interaction energy to the zero time-step limit, as reported in SM. The extrapolated interaction energy with the T-move scheme is − 31.14 ± 2.57 kcal mol^−1^ using LDA nodal structure and − 29.16 ± 2.33 kcal mol^−1^ using PBE0 nodal structure. Due to the large stochastic error on these results, we report the better converged DLA-based interaction energy (with PBE0 nodal structure) in the main results, but we note that all three predictions from FN-DMC agree within the statistical error bars. Furthermore, as the DLA is less sensitive to the Jastrow factor at finite time-steps, we have also tested the interaction energies of GGG, C3A, and C2C2PD complexes, finding agreement with the LA-based FN-DMC results within one standard deviation. Further details can be found in the SM.

The initial DFT orbitals (which define the nodal structure in FN-DMC) were prepared using PWSCF in Quantum Espresso v.6.1^96^ with a plane-wave energy cut-off of 500 Ry. The plane-wave representation of the molecular orbitals from PWSCF were expanded in terms of B-splines. Since PWSCF uses periodic boundary conditions, all complexes were centered in an orthorhombic unit cell with a vacuum spacing of ~ 8 Å in each Cartesian direction to ensure that the single-particle orbitals are fully enclosed. LDA orbitals were used for L7 complexes and in addition, PBE0 orbitals were also considered for C2C2PD, C3GC, and C_60_@[6]CPPA. In all cases, the final FN-DMC interaction energy from LDA and PBE0 nodal structures are in agreement within the stochastic errors.

FN-DMC evaluations of the interaction energy in nine complexes in the S66 set, entries from 24 to 29 and from 47 to 49, were performed with a similar setup. We used the latest version of the Trail and Needs pseudopotentials^97^, and we employed the DLA approximation. LDA orbitals were used for the wave function ansatz, but PBE and PBE0 orbitals were also tested on the benzene dimer (see SM).

## Supplementary information

Supplementary Information

Peer Review File

## Data Availability

The data supporting the findings of this study are available within the paper and its supplementary material. Primary numerical data, e.g., CCSD(T) or FN-DMC energies of molecules are available from PRN and YSA upon reasonable request.
