# Supplementary material for: Interactions between large molecules pose a puzzle for reference quantum mechanical methods
Source: Nat Commun. 2021 Jun 24;12:3927. doi: 10.1038/s41467-021-24119-3 (PMC8225865; doi:10.1038/s41467-021-24119-3)
Supplement: Supplementary file 1 — Supplementary Information [file 41467_2021_24119_MOESM1_ESM.pdf]

Supplemental Material for: Interactions between Large Molecules Pose a Puzzle for Reference Quantum Mechanical Methods

Yasmine S. Al-Hamdani[‡]
Department of Chemistry, University of Zurich, CH-8057 Zürich, Switzerland and
Department of Physics and Materials Science, University of Luxembourg, L-1511 Luxembourg, Luxembourg

Péter R. Nagy‡
Department of Physical Chemistry and Materials Science,
Budapest University of Technology and Economics, H-1521 Budapest, P.O.Box 91, Hungary

Andrea Zen
Dipartimento di Fisica Ettore Pancini, Università di Napoli Federico II, Monte S. Angelo, I-80126 Napoli, Italy
Department of Earth Sciences, University College London,
Gower Street, London WC1E 6BT, United Kingdom and
Thomas Young Centre and London Centre for Nanotechnology,
17-19 Gordon Street, London WC1H 0AH, United Kingdom University

Dennis Barton
Department of Physics and Materials Science, University of Luxembourg, L-1511 Luxembourg, Luxembourg

Mihály Kállay
Department of Physical Chemistry and Materials Science,
Budapest University of Technology and Economics, H-1521 Budapest, P.O.Box 91, Hungary

Jan Gerit Brandenburg[*]
Merck Data Office, Merck KGaA, Frankfurter Str. 250, 64293 Darmstadt, Germany

Alexandre Tkatchenko[†]
Department of Physics and Materials Science, University of Luxembourg, L-1511 Luxembourg, Luxembourg and
[‡] *These authors contributed equally.*

CONTENTS

SUPPLEMENTARY NOTE 1. Details of CCSD(T) computations 2
A. Convergence of local approximations 2
B. Single-particle basis set convergence 3

SUPPLEMENTARY NOTE 2. Details of Quantum Monte Carlo calculations 5
A. Variational Monte Carlo Optimization of the Jastrow Factor 5
B. Time-Step and Node-Structure Dependence of the Coronene Dimer and Benzene Dimer 5
C. The GGG Trimer and Coronene Dimer with the Determinant Localization Approximation 6
D. FN-DMC with T-move on the C_{60}@[6]CPPA Complex 7

SUPPLEMENTARY NOTE 3. Details of DFT calculations 8

SUPPLEMENTARY NOTE 4. Geometry of the L7 and the C_{60}@[6]CPPA complexes 8

Supplementary References 11

[*] j.g.brandenburg@gmx.de
[†] alexandre.tkatchenko@uni.lu

SUPPLEMENTARY NOTE 1. DETAILS OF CCSD(T) COMPUTATIONS

Definitions:

- Interaction energy: according to the Methods Section of the main text, the difference of the complex's energy consisting of all molecules and of the two subsystem energies, using unrelaxed structures for the latter. Notation: $E_{\mathrm{Y}}^{\mathrm{LNO-CCSD(T)}}$[aug-cc-pVXZ], where Y refers to the level of local approximations (*Normal, Tight*, or *very Tight*) and X labels the cardinal number of the basis set.

- counterpoise (CP) corrected interaction energy: the energy of the subsystems are evaluated for the interaction energy expression using all single-particle basis functions of the complete complex including basis functions residing on the atomic positions of the other subsystem.

- local error bar: difference of the Tight and very Tight LNO-CCSD(T) results evaluated with the largest possible basis set.

- basis set incompleteness (BSI) error bar: maximum of two BSI error indicators, which are the difference of the CP corrected and uncorrected LNO-CCSD(T)/CBS(Q,5) interaction energies, and the difference of CP corrected LNO-CCSD(T)/CBS(T,Q) and LNO-CCSD(T)/CBS(Q,5) interaction energies.

A. Convergence of local approximations

The LNO-CCSD(T) energy expression reformulates the CCSD(T) energy in terms of localized molecular orbitals (LMOs, i', j') [1–3]:

$$E^{\mathrm{LNO-CCSD(T)}} = \sum_{i'} \left[\delta E_{i'}^{\mathrm{CCSD(T)}} + \Delta E_{i'}^{\mathrm{MP2}} + \frac{1}{2} \sum_{j'}^{\mathrm{distant}} \delta E_{i'j'} \right]. \tag{1}$$

The correlation energy contribution of distant LMO pairs is obtained at the level of approximate MP2 [3, 4] (third term), while all remaining LMO-pairs contribute to the CCSD(T) level treatment (first term). For the latter, first, local natural orbitals (LNOs) are constructed individually for each LMO at the MP2 level using a large domain of atomic and correlating (virtual) orbitals surrounding the LMO. The $\delta E_{i'}^{\mathrm{CCSD(T)}}$ contribution is then computed in this compressed LNO orbital space, while the second term of Eq. (1) represents a correction for the truncation of the LNO space at the MP2 level of theory.

The convergence of all approximations in LNO-CCSD(T) can be assessed via the use of pre-defined threshold sets, which provide systematic improvement simultaneously for all approximations of the LNO scheme [1–7]. In this series of threshold sets (*Normal*, *Tight*, *very Tight*), the accuracy determining cutoff parameters are tightened in an exponential manner [7]. For instance, the *very Tight* set collects an order of magnitude tighter truncation thresholds than those of the *Normal* set, which is the default choice. The convergence behavior of the LNO-CCSD(T) interaction energies separates the studied complexes (see Fig. 2 of manuscript) into two groups. For GGG, PHE, CBH, and GCGC we observe rapid convergence toward the corresponding canonical CCSD(T) interaction energy as indicated, *e.g.* by the local error estimates collected in Supplementary Table 1. The excellent convergence is apparent as the differences of the *Tight* and *very Tight* interaction energies are all in the 0.1-0.3 kcal mol^{-1} range for these four complexes. This uncertainty range is highly satisfactory for the local approximations considering that the estimated basis set incompleteness (BSI) errors for LNO-CCSD(T) are also comparable.

Supplementary Table 1: Best converged [*Tight–very Tight* extrapolated LNO-CCSD(T)/CBS(Q,5) based] CCSD(T) interaction energies (IEs) and corresponding error estimates with full, half, and no CP correction. Our best estimates are highlighted in bold and are used throughout the manuscript.

System:	GGG	CBH	GCGC	C3A	C2C2PD	PHE	C3GC	C_{60}@[6]CPPA
IE, no CP	-1.98	-10.93	-13.38	-16.79	-20.36	-25.34	-28.67	-41.60
IE, CP	-2.20	-11.10	-13.80	-16.28	-20.84	-25.38	-28.73	-41.89
IE, half CP	**-2.09**	**-11.01**	**-13.59**	**-16.53**	**-20.60**	**-25.36**	**-28.70**	**-41.74**
Local error	0.09	0.10	0.16	0.42	0.38	0.07	0.65	1.10
BSI error	0.11	0.06	0.22	0.24	0.24	0.12	0.19	0.36
Δ_{BSI} error				0.10			0.17	0.25
Total error	0.20	0.15	0.39	0.75	0.62	0.18	1.01	1.71

Consequently, we perform an even more thorough analysis of the local errors for the remaining four complexes, C2C2PD, C3A, C3GC, and C_{60}@[6]CPPA, where the local error estimate of the LNO-CCSD(T) interaction energies is larger than 0.3 kcal mol^{-1}. The convergence patter with *Normal*, *Tight*, and *very Tight* settings of the C3A and C3GC interaction energies is shown on the panel b) of Fig. 4 of the main text. The monitored convergence is monotonic and the remaining local error is about halved in each step, as observed for multiple systems previously [7] as well for the above four complexes. Additionally, the *Normal–Tight* and the *Tight–very Tight* based CCSD(T) estimates (data points with error bars on panel b) of Fig. 4 of the main text) agree closely, and the *Tight–very Tight* error bars are enveloped by the *Normal–Tight* ones. The same trends can be observed in Fig. 1 for the coronene dimer, where LNO-CCSD(T) interaction energies are collected with all investigated basis sets and all three LNO threshold combinations. Again, the convergence patterns with the improving local approximations are parallel for all basis sets, the *Normal–Tight* and *Tight–very Tight* estimates agree within 0.5 kcal mol^{-1}, and the *Tight–very Tight* error bars are 2-3 times narrower. Although, in the case of C_{60}@[6]CPPA, the *Normal* to *very Tight* series is only available with the aug-cc-pVTZ basis set, the 0.2 kcal mol^{-1} agreement of *Normal–Tight* and the *Tight–very Tight* based CCSD(T) estimates and the threefold improvement provided by the *Tight–very Tight* error bar over the *Normal–Tight* one illustrate analogous behavior to the cases of C2C2PD, C3A, and C3GC.

One can also consider internal convergence indicators besides the total energy. At the very Tight level, the π–π, π–σ, and also the majority of the σ–σ orbital interactions benefit from the full CCSD(T) treatment for all complexes. Additionally, none of the remaining weak electronic interactions, contributing only about 0.01% or lower portion of the correlation energy, are neglected, they are, however, approximated via second-order pair energies [3, 7]. At the very Tight level, the orbital domains employed for the LNO-CCSD(T) treatment include all atoms, all atomic orbitals, and the majority of the correlating (virtual) space, spanned by, on the average, 80–95% of the orbitals of the entire complexes.

Finally, the weakly-correlated character of the studied system is also verified via the T1 [8] diagnostics. The T1 measures obtained for the most complicated C3GC and C_{60}@[6]CPPA complexes are found to be at most 0.016 and 0.014, respectively. Considering that the T1 measure grows with the number of basis functions and that smaller than 0.02 T1 values are considered weakly-correlated already for very small systems [8], there appears to be no indication of even moderate static correlation. Moreover, neither the HF nor the CCSD iterations indicated any problems emerging usually for strongly correlated systems. The size of the singles and doubles amplitudes were also monitored in all domain CCSD computations indicating the validity of the single-reference approach, while it is convincing that the LNO approximations were found to operate excellently also for moderately statically correlated species [9]. The magnitude of the (T) correction compared to the full CCSD(T) interaction energy is also an informative measure of the static or dynamic nature of the correlation.

B. Single-particle basis set convergence

Regarding the convergence of the interaction energies with respect to the single particle basis set, we rely on approaches used routinely in wavefunction computations on small molecules. Dunning's correlation consistent basis sets [10] employed here are designed to systematically approach the complete basis set (CBS) limit with a polynomial convergence rate, which can be exploited to reduce the remaining basis set incompleteness (BSI) error via basis set extrapolation approaches [11, 12]. We employ two-point formulae for extrapolation, labeled as CBS(X,$X+1$), where X refers to the cardinal number of the aug-cc-pVXZ basis set [10] with X=T, Q, and 5.

For the proper description of important medium- and long-range interactions and of the cross-polarization of the monomers in the complex, it is crucial to the employ diffuse, i.e., spatially spread basis functions. The use of such

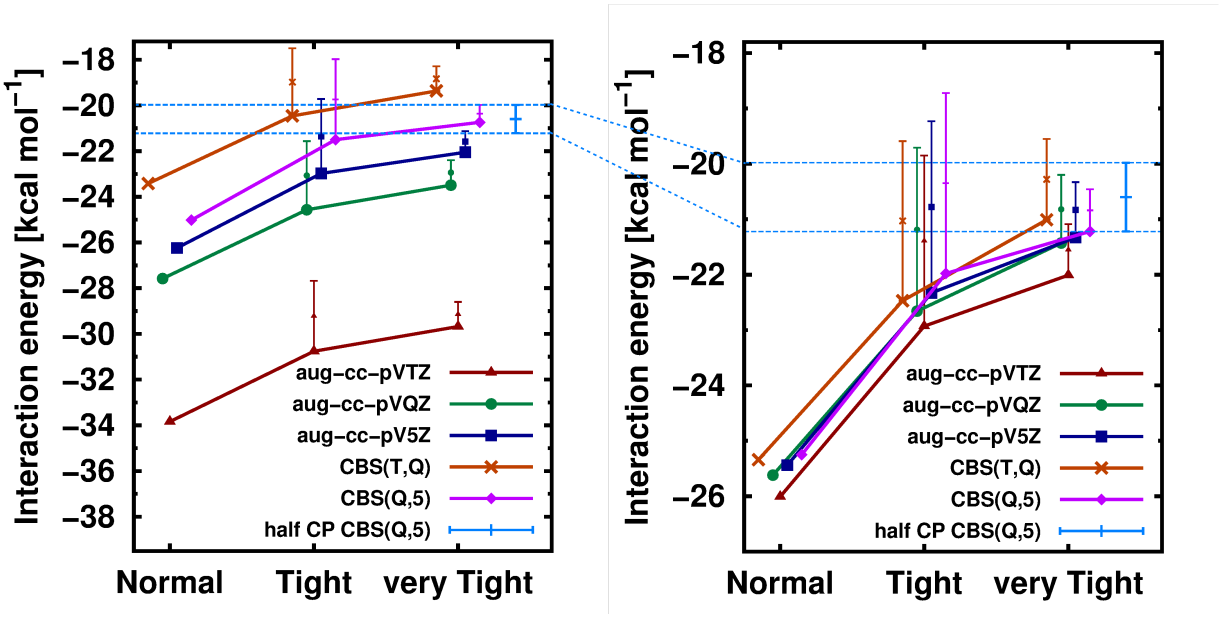

Supplementary Fig. 1: Convergence of LNO-CCSD(T) interaction energies for the C2C2PD complex with various basis sets and LNO threshold sets. The left (right) panel collects results obtained without (with) CP correction. The *Normal*–*Tight* and the *Tight*–*very Tight* extrapolated results are plotted with a smaller point size also at the *Tight* and *very Tight* x axis labels, respectively, and they are accompanied by error bars indicating the uncertainty estimate of the local approximations at that level. For comparison the best CCSD(T)/CBS estimate [Tight-very Tight approximated, half CP corrected LNO-CCSD(T)/CBS(Q,5)] result and its corresponding uncertainty estimate is depicted on both panels via the light blue error bars and dashed horizontal lines. Note the different y ranges of the two panels as highlighted by dashed blue lines connecting the two panels. Also note that symbols corresponding to a given basis set are slightly shifted along the x-axis to improve visibility for all data points.

diffuse basis functions, however, greatly enhances technical challenges characteristic of interaction energy computations with atom centered Gaussian type basis functions. As long as the basis set expansion of the monomers is not saturated completely, the basis functions residing on the atoms of one monomer can contribute to the description of the wavefunction components of the other monomer. Thus, the resulting basis set superposition error (BSSE) emerges from the unbalanced improvement of the basis set expansion of the monomers and the dimer and usually leads to artificially overestimated interaction strength. The BSSE can be decreased significantly by counterpoise (CP) corrections [13], i.e., by using the entire dimer basis set also for the monomer calculations. Naturally, for small basis sets this approach might lead to a more saturated basis set expansion on the monomers and can potentially overcorrect the BSSE. In the case of aug-cc-pVXZ with X=T, Q, and 5 the CP correction decreases monotonically with increasing basis set size, thus a decreasing CP correction is an excellent indicator of basis set saturation, which we employ here.

To characterize the convergence of the LNO-CCSD(T) interaction energies in terms of the basis set completeness, the maximum of two BSI error indicators is considered with the best available LNO threshold set. One of them is the difference of the CP corrected and uncorrected LNO-CCSD(T)/CBS(Q,5) interaction energies, and the other one is the difference of CP corrected LNO-CCSD(T)/CBS(T,Q) and LNO-CCSD(T)/CBS(Q,5) interaction energies. The resulting BSI error bar values of Supplementary Table 1 indicate that the above two four-membered groups exhibit much more homogeneous basis set convergence behavior. For the GGG, GCGC, PHE, and CBH interaction energies, this BSI measure is 0.06-0.22 kcal mol^{-1}, while for the other four complexes a twice as large uncertainty of 0.19-0.36 kcal mol^{-1} is found. Compared to the similar or larger local error bars, we find this level of basis set convergence to be highly satisfactory.

We again investigate more closely only the C3A, C3GC, C2C2PD, and C_{60}@[6]CPPA quartet. The convergence of LNO-CCSD(T) interaction energies with improving basis sets for C3A and C3GC is shown on panel a) of Fig. 4 of the main text. The large BSSE obtained with the aug-cc-pVTZ, and to some extent also with the aug-cc-pVQZ basis set is apparent for both complexes. Such large BSSE also affects the extrapolation, the CBS(T,Q) results clearly overshoot the basis set limit due to the underestimation of the aug-cc-pVTZ result. The BSSE is significantly reduced by the CP correction. All CP corrected results (solid symbols) closely agree already at the aug-cc-pVTZ level. Most

importantly, the CBS(Q,5) entries of both the CP corrected and uncorrected series match each other within a few tenth of a kcal mol^{-1}, providing strong indication of basis set saturation. Upon inspection of the CP corrected and uncorrected interaction energies of Supplementary Table 1, this statement can be extended for the remaining six complexes as well.

The left and right panels of Supplementary Fig. 1 collect CP uncorrected and corrected LNO-CCSD(T) interaction energies for the coronene dimer. The overbinding of the aug-cc-pVTZ and aug-cc-pVQZ results caused by the BSSE is again significant, close to 50 and 20%, respectively. With the exception of the overshooting CBS(T,Q) extrapolation, the aug-cc-pVXZ energies, with X=T, Q, and 5, as well as the CBS(Q,5) extrapolation form a highly convincing, converging series of results both with and without CP correction. The CP corrected and uncorrected CBS results approach the region of convergence from the opposite directions, hence their average, i.e., the half CP corrected results appear to be the best estimate at the CBS(Q,5) level. Concerning CBS(T,Q), the fully CP corrected results are found more reliable due to the excessive BSSE obtained with aug-cc-pVTZ.

Finally, we assess the accuracy of the composite BSI correction approach employed for C3A, C3GC, and C_{60}@[6]CPPA. Due to the prohibitive computational costs, the most accurate interaction energies presented here for these three systems are obtained by adding a $\Delta_{\rm BSI} = E^{\rm LNO-CCSD(T)}_{\rm Normal}[{\rm CBS(Q,5)}] - E^{\rm LNO-CCSD(T)}_{\rm Normal}[\text{aug-cc-pVTZ}]$ BSI correction to the $E^{\rm LNO-CCSD(T)}_{\rm Tight-very\ Tight}[\text{aug-cc-pVTZ}]$ interaction energies. This formula exploits the similarity of the local approximation convergence curves obtained with different basis sets and it is numerically identical to $E^{\rm LNO-CCSD(T)}_{\rm Tight-very\ Tight}[{\rm CBS(Q,5)}]$ if the local convergence patterns are exactly parallel. To assess the quality of $\Delta_{\rm BSI}$, we compared $\Delta_{\rm BSI}$ to the analogous $\Delta^{\rm very\ Tight}_{\rm BSI} = E^{\rm LNO-CCSD(T)}_{\rm very\ Tight}[{\rm CBS(Q,5)}] - E^{\rm LNO-CCSD(T)}_{\rm very\ Tight}[\text{aug-cc-pVTZ}]$ wherever it is available. For the system most similar with the above three, that is, for C2C2PD, the $|\Delta^{\rm very\ Tight}_{\rm BSI} - \Delta_{\rm BSI}|$ value is about 0.12 kcal mol^{-1}. To account for the potentially size-extensive nature of this unparallelity error, the final $\Delta_{\rm BSI}$ error estimates of Supplementary Table 1 were obtained by scaling the 0.12 kcal mol^{-1} with the ratio of the interaction energies of the given complex and C2C2PD. The "Total error bar" values of Supplementary Table 1 also include this third, $\Delta_{\rm BSI}$ related uncertainty estimate for these three complexes.

SUPPLEMENTARY NOTE 2. DETAILS OF QUANTUM MONTE CARLO CALCULATIONS

The FN-DMC calculations mostly used 10 nodes with 28 cores each, and 14,000 walkers distributed across the cores (*i.e.* 50 walkers per core). We used 20 nodes for the C_{60}@[6]CPPA complex and 28,000 walkers to reduce the stochastic error in a shorter time. Here we give further details on (i) the optimization of the Jastrow factor for the reported complexes, (ii) time-step and node-structure tests for the coronene dimer and (iii) results of additional FN-DMC simulations of C_{60}@[6]CPPA. Typically, the reported error bar in FN-DMC corresponds to one standard deviation (σ). The $\pm\sigma$ interval corresponds to less than 70%. This is quite low for a meaningful comparison with other approaches, therefore we opted for an an interval of 95% in the main results while we show the typical $\pm\sigma$ interval here in the supplemental.

A. Variational Monte Carlo Optimization of the Jastrow Factor

Variational Monte Carlo (VMC) obeys the variational principle, allowing the initial Slater-Jastrow wavefunction to be optimized iteratively towards a lower energy. Importantly, the zero-variance principle ensures that variance of the energy tends to zero as the exact energy of the system is approached. This is used in the varmin and varmin–linjas optimization algorithms in CASINO [14] to optimize the variable parameters of the Jastrow factor. The Jastrow factor is composed of explicit distance-dependent polynomial functions for inter-particle interactions, such as electron-electron (u), electron-nucleus (χ), and electron-electron-nucleus (f), and is also system-dependent. For all complexes, we performed a term-by-term optimization using 24 parameters for u, 12-14 parameters per element for χ, and 8 parameters per element for f. The resulting VMC energy and variance for the complexes is given in Supplementary Table 2.

B. Time-Step and Node-Structure Dependence of the Coronene Dimer and Benzene Dimer

It can be seen from Supplementary Fig. 2 that the FN-DMC interaction energy of C2C2PD is converged within the stochastic error bar (corresponding to 1 standard deviation) with respect to the time-step in FN-DMC (from 0.003 to 0.03 a.u.). In addition, we computed PBE0 and PBE initial determinants (orbitals) from PWSCF, in order to assess the FN-DMC dependence of the interaction energy on the nodal-structure. An unconstrained propagation of

Supplementary Table 2: The variance (σ^2) and VMC energy (E_{VMC}) in atomic units for each complex, as a result of optimizing the trial wavefunction. The uncertainty is indicated in parentheses.

Complex	σ^2	E_{VMC}
CBH	4.07(5)	-249.26(1)
C2C2PD	4.76(6)	-285.769(8)
GGG	5.18(3)	-290.195(5)
GCGC	5.71(3)	-336.296(1)
PHE	6.03(4)	-367.239(8)
C3A	6.38(4)	-397.085(6)
C3GC	7.82(5)	-484.474(7)
C_{60}@[6]CPPA	10.54(5)	-624.140(6)

an electronic wavefunction would project out a bosonic function, which is an unphysical solution. To prevent this, the only affordable solution for large molecular systems is to constrain the walkers to not cross the nodal surface. In this way the projected wave function Ψ_{FN} has the same nodes as the trial wave function Ψ_T.

Supplementary Fig. 2 shows that the FN-DMC interaction energy is the same within the stochastic error bars of ~0.5 kcal mol^{-1} across the three nodal-structures.

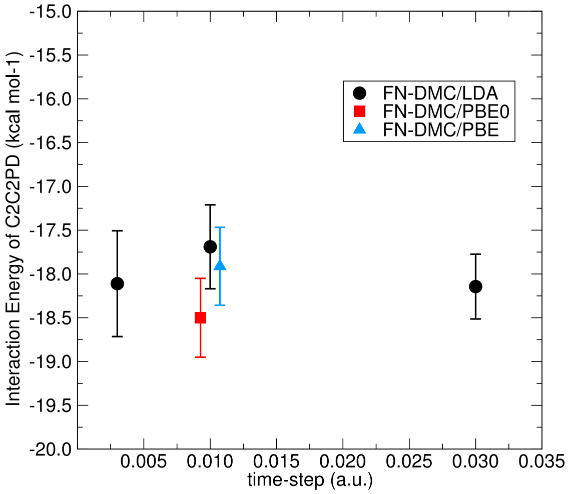

Supplementary Fig. 2: FN-DMC interaction energy of C2C2PD (coronene dimer) with 0.003, 0.01 and 0.03 a.u. time-steps. Different nodal-structures from LDA (black circle), PBE (blue triangle), and PBE0 (red square) initial orbitals are reported using 0.01 a.u. time-step; these are slightly offset along the x-axis for clarity. Here the stochastic uncertainty corresponds to 1-σ.

Similar considerations apply also to the parallel displaced benzene dimer, entry 24 in the S66 set. Supplementary Fig. 3 shows the DMC evaluations of the interaction energy as obtained with different time-steps and different initialisations of the orbitals. It appears that any possible bias given by the orbitals is much smaller than the stochastic uncertainty of the DMC evaluations. Moreover, a time-step of 0.03 or smaller has a negligible bias compared to the stochastic uncertainty. This conclusion applies as well to all the other 8 complexes of the S66 set that we considered in this work (24 to 29, and 47 to 49). In the manuscript we report the results obtained with a time-step of 0.01 a.u.

We note that the agreement between FN-DMC and CCSD(T) on small-to-medium sized dimers is in-line with previous predictions of carbon dioxide, ammonia, benzene, anthracene, and naphthalene dimer interaction energies as shown in the supporting information of Ref. [15].

C. The GGG Trimer and Coronene Dimer with the Determinant Localization Approximation

Using non-local pseudopotentials in FN-DMC requires an approximation for the evaluation of the local energy – not to be confused with the type of local approximations, such as LNO, made in local CCSD(T) methods. The recent

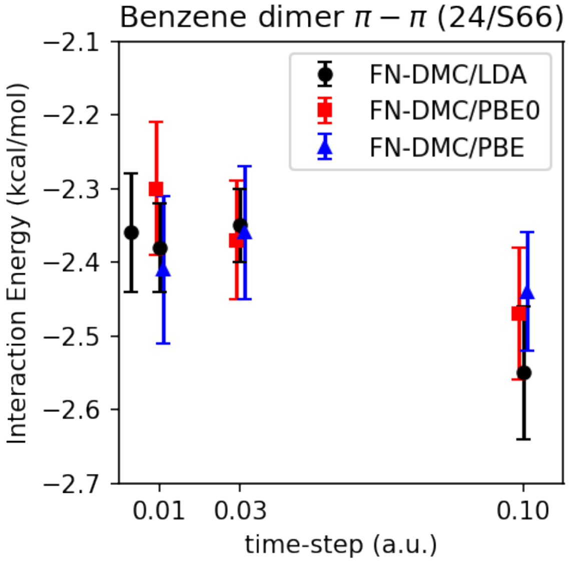

Supplementary Fig. 3: FN-DMC interaction energy of the benzene dimer parallel displaced (entry 24 in the S66 set). Different nodal-structures from LDA (black circle), PBE (blue triangle), and PBE0 (red square) initial orbitals are reported using 0.003 (only for LDA), 0.01, 0.03 and 0.1 a.u. time-step; these are slightly offset along the x-axis for clarity. The error bars account for the stochastic uncertainty of the DMC estimations and correspond here to 1 standard deviation (i.e. a 95% confidence interval is roughly twice as large as the reported error bars).

determinant localization approximation (DLA) introduced by Zen *et al.* [16] has some advantages over the pre-existing standard algorithms: the locality approximation [17] (LA) and T-move scheme [18]). The DLA FN-DMC energies are less sensitive to the Jastrow factor that is used in combination with pseudopotentials at larger time-steps. This enables better overall convergence with the time-step in FN-DMC and the DLA method is also more numerically stable than LA. We tested the use of the DLA method for the GGG trimer and the coronene dimer and present the results in Supplementary Table 3. The interaction energies of the GGG and C2C2PD complexes remain in agreement,

Supplementary Table 3: Comparison of the standard LA to the DLA method in the GGG and C2C2PD complexes with 1-σ stochastic uncertainty.

Complex	Approximation	Time-step	IE (kcal mol^{-1})
GGG	standard LA	0.03	1.5 ± 0.3
GGG	DLA	0.03	1.4 ± 0.2
C2C2PD	standard LA	0.03	-18.1 ± 0.4
C2C2PD	DLA	0.01	-17.4 ± 0.5

within the one-standard deviation stochastic errors, between the DLA and the standard LA algorithms. The results support that the FN-DMC results are converged with respect to the time-steps and employed Jastrow factors.

D. FN-DMC with T-move on the C_{60}@[6]CPPA Complex

The C_{60}@[6]CPPA complex proved to be more challenging to compute with FN-DMC, due to numerical instabilities when using the locality approximation. This was alleviated by the use of the DLA method, and separately using the T-move approximation in place of the locality approximation. The T-move scheme reinstates variational form of the energy, but the energies with this approximation are more time-step dependent, as can be seen in Supplementary Fig. 4. Linear extrapolations to 'zero' time-step limit yield -31.14 ± 2.57 kcal mol^{-1} using LDA orbitals and -29.16 ± 2.33 kcal mol^{-1} using PBE0 orbitals (with 1-σ stochastic uncertainty). Moreover, we show the DLA obtained FN-DMC interaction energy at 0.03 and 0.01 a.u. time-steps. In this way, the independence of the interaction energy on the nodal structure and the FN-DMC algorithm is established.

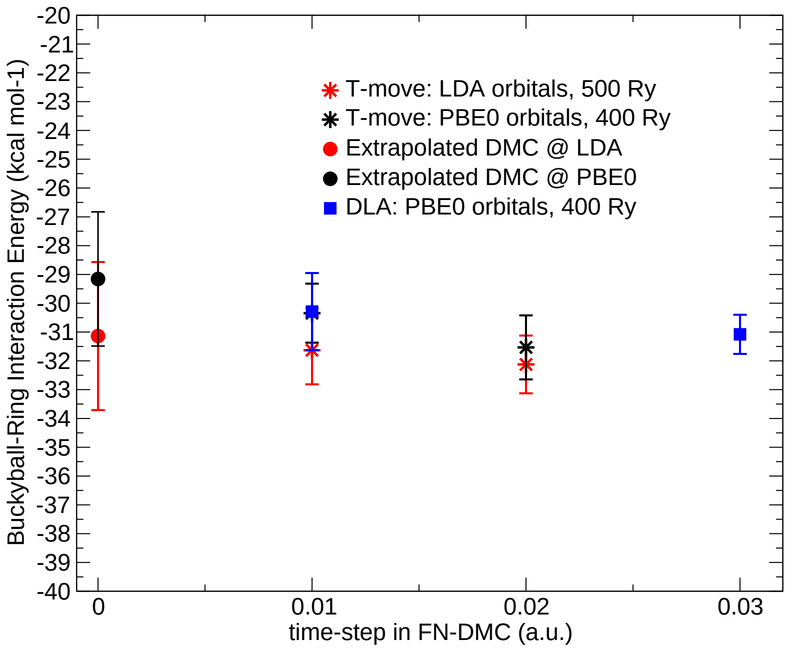

Supplementary Fig. 4: FN-DMC interaction energy of C_{60}@[6]CPPA complex using two algorithms. T-move interaction energies at 0.01 and 0.02 a.u. time-steps are shown for LDA (black stars) and PBE0 orbitals (red stars). The linear extrapolation to zero time-step for each set is indicated by the dashed lines, with the result in circles. The error on the zero time-step FN-DMC interaction energies are propagated according to the extrapolation from the 1-σ error bars. For comparison, the DLA method is shown in blue squares. The DLA FN-DMC interaction energy at 0.01 a.u. is slightly offset along the x-axis for clarity.

SUPPLEMENTARY NOTE 3. DETAILS OF DFT CALCULATIONS

The PBE0+MBD calculations were performed using FHI-aims v.190225 with all-electron numerical basis sets, with "tight" defaults and tier 2 basis functions for all elements. The total energy threshold for self-consistent convergence was set to 10^{-7} eV. Spin and relativistic effects have not been included. London dispersion energies from the D4 model are computed with the DFTD4 standalone program using the electronegativity equilibration charges (EEQ) and include a coupled-dipole based many-body dispersion correction (D4(EEQ)-MBD) [19]. The same geometries have been used as for the benchmark calculations for all structures.

SUPPLEMENTARY NOTE 4. GEOMETRY OF THE L7 AND THE C_{60}@[6]CPPA COMPLEXES

The structures and fragment definitions in Ref. 20 were used for the L7 calculations. For C_{60}@[6]CPPA, a C_{70}@[6]CPPA geometry from Ref. 21 was modified, by replacing C_{70} with C_{60} and the complex was symmetrized to D_{3d} point group. The high-symmetry structure allows more efficient calculations with LNO-CCSD(T) with a speedup proportional to the rank of the point group [3, 5]. The stability of this complex was assessed by relaxing the geometry whilst retaining the symmetry group, at the DFT level (B97-3c exchange-correlation functional). The interaction strength increases by less than 0.1 kcal mol^{-1} with respect to the unrelaxed structure. Relaxing the C_{60} and [6]CPPA fragments reduces the interaction strength by 0.9 kcal mol^{-1}.

The C_{60}@[6]CPPA Cartesian coordinates used in LNO-CCSD(T) and FN-DMC calculations is given here.

```
C       -0.72650728     -1.22225849     -3.24715547
C        0.72650728     -1.22225849     -3.24715547
C       -1.42176054     -0.01804451     -3.24715547
```

C	1.42176054	-0.01804451	-3.24715547
C	2.59727407	0.14217202	-2.40825772
C	1.17551245	-2.32039134	-2.40825772
C	2.30045705	-2.16706760	-1.60544793
C	3.02696412	-0.90872044	-1.60544793
C	-3.02696412	-0.90872044	-1.60544793
C	-2.30045705	-2.16706760	-1.60544793
C	-2.59727407	0.14217202	-2.40825772
C	-1.17551245	-2.32039134	-2.40825772
C	0.00000000	-2.99907290	-1.88979043
C	-2.30045914	-2.68553418	-0.24808191
C	-1.17551125	-3.33502315	0.24808191
C	0.00000000	-3.49523729	-0.59081634
C	-3.02696429	1.74761865	-0.59081634
C	-3.47597040	0.64948897	0.24808191
C	-2.59727332	1.49953645	-1.88979043
C	-3.47597040	-0.64948897	-0.24808191
C	-3.02696429	-1.74761865	0.59081634
C	-3.02696412	0.90872044	1.60544793
C	-2.59727407	-0.14217202	2.40825772
C	-2.59727332	-1.49953645	1.88979043
C	-0.72650707	3.07578804	-1.60544793
C	-1.17551125	3.33502315	-0.24808191
C	-1.42176162	2.17821932	-2.40825772
C	-2.30045914	2.68553418	0.24808191
C	-2.30045705	2.16706760	1.60544793
C	-0.00000000	3.49523729	0.59081634
C	-0.00000000	2.99907290	1.88979043
C	-1.17551245	2.32039134	2.40825772
C	0.69525326	1.24030300	-3.24715547
C	1.42176162	2.17821932	-2.40825772
C	-0.69525326	1.24030300	-3.24715547
C	0.72650707	3.07578804	-1.60544793
C	1.17551125	3.33502315	-0.24808191
C	2.59727332	1.49953645	-1.88979043
C	3.02696429	1.74761865	-0.59081634
C	2.30045914	2.68553418	0.24808191
C	0.72650728	1.22225849	3.24715547
C	1.17551245	2.32039134	2.40825772
C	2.30045705	2.16706760	1.60544793
C	1.42176054	0.01804451	3.24715547
C	-0.69525326	-1.24030300	3.24715547
C	-1.42176054	0.01804451	3.24715547
C	-0.72650728	1.22225849	3.24715547
C	0.69525326	-1.24030300	3.24715547
C	0.72650707	-3.07578804	1.60544793
C	-0.72650707	-3.07578804	1.60544793
C	-1.42176162	-2.17821932	2.40825772
C	1.42176162	-2.17821932	2.40825772
C	3.02696429	-1.74761865	0.59081634
C	2.30045914	-2.68553418	-0.24808191
C	1.17551125	-3.33502315	0.24808191
C	2.59727332	-1.49953645	1.88979043
C	3.02696412	0.90872044	1.60544793
C	3.47597040	0.64948897	0.24808191
C	3.47597040	-0.64948897	-0.24808191
C	2.59727407	-0.14217202	2.40825772
C	-4.43498968	4.84818396	-0.00326334

C	-5.43089278	3.84285784	-0.00366147
C	-3.85193459	5.28074380	-1.21899357
C	-3.85171966	5.27950986	1.21280412
C	-2.64632983	5.97544200	-1.21280412
C	-2.64729099	5.97624511	1.21899357
C	-1.98115563	6.26490571	0.00326334
C	-6.04345890	2.78186219	-0.00366147
H	-4.31742848	4.99255327	-2.16174586
H	-4.31701338	4.99032575	2.15534928
H	-2.16324219	6.23380613	-2.15534928
H	-2.16496372	6.23527938	2.16174586
C	1.98115563	6.26490571	0.00326334
C	0.61256612	6.62472003	0.00366147
C	2.64632983	5.97544200	-1.21280412
C	2.64729099	5.97624511	1.21899357
C	3.85193459	5.28074380	-1.21899357
C	3.85171966	5.27950986	1.21280412
C	4.43498968	4.84818396	-0.00326334
C	-0.61256612	6.62472003	0.00366147
H	2.16324219	6.23380613	-2.15534928
H	2.16496372	6.23527938	2.16174586
H	4.31742848	4.99255327	-2.16174586
H	4.31701338	4.99032575	2.15534928
C	6.41614531	1.41672175	-0.00326334
C	6.04345890	2.78186219	-0.00366147
C	6.49922558	0.69550131	-1.21899357
C	6.49804949	0.69593214	1.21280412
C	6.49804949	-0.69593214	-1.21280412
C	6.49922558	-0.69550131	1.21899357
C	6.41614531	-1.41672175	0.00326334
C	5.43089278	3.84285784	-0.00366147
H	6.48239220	1.24272611	-2.16174586
H	6.48025557	1.24348038	2.15534928
H	6.48025557	-1.24348038	-2.15534928
H	6.48239220	-1.24272611	2.16174586
C	4.43498968	-4.84818396	0.00326334
C	5.43089278	-3.84285784	0.00366147
C	3.85171966	-5.27950986	-1.21280412
C	3.85193459	-5.28074380	1.21899357
C	2.64729099	-5.97624511	-1.21899357
C	2.64632983	-5.97544200	1.21280412
C	1.98115563	-6.26490571	-0.00326334
C	6.04345890	-2.78186219	0.00366147
H	4.31701338	-4.99032575	-2.15534928
H	4.31742848	-4.99255327	2.16174586
H	2.16496372	-6.23527938	-2.16174586
H	2.16324219	-6.23380613	2.15534928
C	-1.98115563	-6.26490571	-0.00326334
C	-0.61256612	-6.62472003	-0.00366147
C	-2.64729099	-5.97624511	-1.21899357
C	-2.64632983	-5.97544200	1.21280412
C	-3.85171966	-5.27950986	-1.21280412
C	-3.85193459	-5.28074380	1.21899357
C	-4.43498968	-4.84818396	0.00326334
C	0.61256612	-6.62472003	-0.00366147
H	-2.16496372	-6.23527938	-2.16174586
H	-2.16324219	-6.23380613	2.15534928
H	-4.31701338	-4.99032575	-2.15534928

H	-4.31742848	-4.99255327	2.16174586
C	-6.41614531	-1.41672175	0.00326334
C	-6.04345890	-2.78186219	0.00366147
C	-6.49804949	-0.69593214	-1.21280412
C	-6.49922558	-0.69550131	1.21899357
C	-6.49922558	0.69550131	-1.21899357
C	-6.49804949	0.69593214	1.21280412
C	-6.41614531	1.41672175	-0.00326334
C	-5.43089278	-3.84285784	0.00366147
H	-6.48025557	-1.24348038	-2.15534928
H	-6.48239220	-1.24272611	2.16174586
H	-6.48239220	1.24272611	-2.16174586
H	-6.48025557	1.24348038	2.15534928

SUPPLEMENTARY REFERENCES

[1] Z. Rolik, L. Szegedy, I. Ladjánszki, B. Ladóczki, and M. Kállay, An efficient linear-scaling CCSD(T) method based on local natural orbitals, J. Chem. Phys. **139**, 094105 (2013).

[2] P. R. Nagy and M. Kállay, Optimization of the linear-scaling local natural orbital CCSD(T) method: Redundancy-free triples correction using Laplace transform, J. Chem. Phys. **146**, 214106 (2017).

[3] P. R. Nagy, G. Samu, and M. Kállay, Optimization of the linear-scaling local natural orbital CCSD(T) method: Improved algorithm and benchmark applications, J. Chem. Theory Comput. **14**, 4193 (2018).

[4] P. R. Nagy, G. Samu, and M. Kállay, An integral-direct linear-scaling second-order Møller–Plesset approach, J. Chem. Theory Comput. **12**, 4897 (2016).

[5] Z. Rolik and M. Kállay, A general-order local coupled-cluster method based on the cluster-in-molecule approach, J. Chem. Phys. **135**, 104111 (2011).

[6] M. Kállay, Linear-scaling implementation of the direct random-phase approximation, J. Chem. Phys. **142**, 204105 (2015).

[7] P. R. Nagy and M. Kállay, Approaching the basis set limit of CCSD(T) energies for large molecules with local natural orbital coupled-cluster methods, J. Chem. Theory Comput. **15**, 5275 (2019).

[8] T. J. Lee and P. R. Taylor, A diagnostic for determining the quality of single-reference electron correlation methods, Int. J. Quantum Chem. **36**, 199 (1989).

[9] N. Sylvetsky, A. Banerjee, M. Alonso, and J. M. L. Martin, Performance of localized coupled cluster methods in a moderately strong correlation regime: Hückel–Möbius interconversions in expanded porphyrins, J. Chem. Theory Comput. **16**, 3641 (2020).

[10] R. A. Kendall, T. H. Dunning Jr., and R. J. Harrison, Electron affinities of the first-row atoms revisited. Systematic basis sets and wave functions, J. Chem. Phys. **96**, 6796 (1992).

[11] A. Karton and J. M. L. Martin, Comment on: "Estimating the Hartree–Fock limit from finite basis set calculations", Theor. Chem. Acc. **115**, 330 (2006).

[12] T. Helgaker, W. Klopper, H. Koch, and J. Noga, Basis-set convergence of correlated calculations on water, J. Chem. Phys. **106**, 9639 (1997).

[13] S. F. Boys and F. Bernardi, The calculation of small molecular interactions by the differences of separate total energies. Some procedures with reduced errors, Mol. Phys. **19**, 553 (1970).

[14] R. J. Needs, M. D. Towler, N. D. Drummond, and P. López Ríos, Continuum variational and diffusion quantum Monte Carlo calculations, J. Phys. Condens. Matter **22**, 023201 (2010).

[15] A. Zen, J. G. Brandenburg, J. Klimeš, A. Tkatchenko, D. Alfè, and A. Michaelides, Fast and accurate quantum Monte Carlo for molecular crystals, Proc. Natl. Acad. Sci. **115**, 1724 (2018).

[16] A. Zen, J. G. Brandenburg, A. Michaelides, and D. Alfè, A new scheme for fixed node diffusion quantum Monte Carlo with pseudopotentials: Improving reproducibility and reducing the trial-wave-function bias, J. Chem. Phys. **151**, 134105 (2019).

[17] L. Mitáš, E. L. Shirley, and D. M. Ceperley, Nonlocal pseudopotentials and diffusion Monte Carlo, J. Chem. Phys. **95**, 3467 (1991).

[18] M. Casula, Beyond the locality approximation in the standard diffusion Monte Carlo method, Phys. Rev. B **74**, 161102 (2006).

[19] E. Caldeweyher, S. Ehlert, A. Hansen, H. Neugebauer, S. Spicher, C. Bannwarth, and S. Grimme, A generally applicable atomic-charge dependent london dispersion correction, J. Chem. Phys. **150**, 154122 (2019).

[20] R. Sedlak, T. Janowski, M. Pitoňák, J. Řezáč, P. Pulay, and P. Hobza, Accuracy of quantum chemical methods for large noncovalent complexes, J. Chem. Theory Comput. **9**, 3364 (2013).

[21] J. Hermann, D. Alfè, and A. Tkatchenko, Nanoscale π-π Stacked molecules are bound by collective charge fluctuations, Nat. Commun. **8**, 14052 (2017).
