# Supplementary material for: Interactions between large molecules pose a puzzle for reference quantum mechanical methods
Source: Nat Commun. 2021 Jun 24;12:3927. doi: 10.1038/s41467-021-24119-3 (PMC8225865; doi:10.1038/s41467-021-24119-3)
Supplement: Supplementary file 2 — Peer Review File [file 41467_2021_24119_MOESM2_ESM.pdf]

Editorial Note: This manuscript has been previously reviewed at another journal that is not operating a transparent peer review scheme. This document only contains reviewer comments and rebuttal letters for versions considered at *Nature Communications*. Mentions of prior referee reports have been redacted.

REVIEWER COMMENTS

Reviewer #4 (Remarks to the Author)

First, I would like to respond to the remark of referee 3 **[REDACTED]** My comment was not just based on size extensivity (defined as approximately linear increase of magnitude of the total energy with system size). There were two important elements of my argument that lead to the conclusion. One is that generally the ratio of the interaction energy to the total energy decreases as system size increases. Second, numerical implementations of electronic structure methods with a given set of accuracy thresholds tend to produce a fixed number of accurate significant digits in total energies. This leads to cancellations of several significant digits in subtractions of the monomer energies from the dimer energy. I also do not say that the discrepancies obtained by the authors are obvious, I only say that discrepancies can be expected to increase when moving to systems with sizes significantly larger than previously investigated.

Referee 2 discusses in detail novelty of the finding of the present work compared to published comparisons of FN-DMC and CCSD(T), including a paper by some of the present authors, Ref. 9. I generally agree with the overall conclusions that that the authors overemphasize the novelty of their findings. Furthermore, although the history of computational chemistry is full of papers showing disagreements between various methods, and I do not remember any of these papers being published in Nature.

The authors made some revisions of the paper, and, with these changes, I mostly agree with what they say, with two exceptions. I still do not agree with the last line of the abstract that states: "Our data contradicts the expectation that the state-of-the-art realizations of the most comprehensive and robust wavefunction methods generally predict identical non-covalent interactions and indicate an unsolved challenge for benchmark approaches." This is not my expectation. Just the opposite, as stated above, I expect that numerical implementations (presumably what the authors mean by "state-of-the-art realizations") of electronic structure methods that agree on smaller systems will agree less well when applied to significantly larger systems. The quoted sentence should simply be removed. Another place that should be modified is the middle paragraph on p. 8, which is constructed in an really convoluted way in order not to say that the paper by some of the present authors, Ref. 9, did report discrepancies between DMC and CCSD(T) for large dimers.

In the second report, referee 2 still finds that the paper should not be published at all, while referee 3 states that the paper should be submitted to **[REDACTED]** Clearly, Nature Communications does not belong to this category. I agree with the recommendation of referee 3, with a minor twist that I would like the authors to modify the statements discussed in the previous paragraph.

Reviewer #5 (Remarks to the Author):

The paper describes a discrepancy between two methods claimed to be of benchmark quality, CCSD(T) and FN-DMC, applied to calculations of interaction energies in larger molecular complexes. The authors show that in some of the studied systems, the difference between the results obtained with these two methods is significantly larger than what can be explained by their estimate of the errors of the method (based on calculations in smaller systems), and they suggest that there is some fundamental difference in how these methods describe small and large molecular systems.

I would, however, argue that the error estimates on which all the discussion and conclusions stand are wrong, at least at the side of FN-DMC. In fact, there is only very little evidence that FN-DMC and CCSD(T) yield comparable results in small and medium-sized systems, and it is not sufficient for making general, statistically relevant conclusions. The authors cite results obtained in the A24 data set which features only very small systems,(Ref. 42) another work on 6 similar (or identical) molecular complexes (Ref. 2), but no systematic study is available on non-covalent interactions in a large- and diverse-enough set of medium-sized complexes where reliable CCSD(T) results are still available (e.g. at least in the S66 data set).

For systems of this size, the authors showcase the benzene--water complex as an example where there is good agreement between CCSD(T) and FN-DMC. On the other hand, they do omit a much more important and thoroughly studied example, the benzene dimer in the parallel-displaced stacked orientation. It is an important model for pi-pi interactions (which comprise a dominant part of the systems studied in the reviewed paper), and there are very accurate benchmark data available at the CCSD(T) level and beyond. Indeed, it has already been studied also by Monte Carlo methods (Azadi and Cohen 2015, Gasperich and Jordan 2016), and the results are considerably worse there. Specifically, in the former paper, FN-DMC was found to yield an error of almost 1 kcal/mol (with CCSD(T) benchmark of -2.65 kcal/mol) that could be eliminated by applying the backflow transformations that lift the limitations imposed by the fixed-node approximation. The second paper also attributes the error to this approximation. Although a different setup may have been used for the FN-DMC calculations in the present study, it is up to the authors to prove its accuracy.

If the accuracy of FN-DMC (without the backflow transformation correction) is estimated using the available results for benzene dimer as the only model, the error amounts to more than 30%, what would easily explain the observations reported in the paper. It also makes the whole discussion of the differences between CSCD(T) and FN-DMC in the present work worthless. Moreover, it is hard to believe that the omission of the benzene dimer is not intentional. It is so important model system (especially when pi-pi interactions are studied) widely studied in the literature, that it would be a glaring negligence. In the other case, it would be a deliberate manipulation that is even worse.

If the authors want to discuss the errors in large systems, they should start with validating the method in medium-sized systems first. Here, a statistically significant number of systems (several tens, including pi-pi complexes such as benzene dimer, stacked nuceobases, etc.) should be computed with exactly the same FN-DMC setup used for the large ones. Until the issues in smaller systems are characterized and resolved, the calculations on large systems are worthless.

On the basis of these findings, I can not recommend the present manuscript for publication neither here, nor in any other journal. It could possibly be pared down to a comparison of results with unknown accuracy on the FN-DMC side if a honest overview of smaller models is included, and such a study would belong to a specialized computational chemistry journal.

Please find below our point by point reply to the Referees' comments made to our revised manuscript.

===== Referees' comments =====

Reviewer 4 (Remarks to the Author)

First, I would like to respond to the remark of referee 3 . [REDACTED] My comment was not just based on size extensivity (defined as approximately linear increase of magnitude of the total energy with system size). There were two important elements of my argument that lead to the conclusion. One is that generally the ratio of the interaction energy to the total energy decreases as system size increases. Second, numerical implementations of electronic structure methods with a given set of accuracy thresholds tend to produce a fixed number of accurate significant digits in total energies. This leads to cancellations of several significant digits in subtractions of the monomer energies from the dimer energy. I also do not say that the discrepancies obtained by the authors are obvious, I only say that discrepancies can be expected to increase when moving to systems with sizes significantly larger than previously investigated.

We replied to this point in agreement with the interpretation of Reviewer 4 and that reply was accepted in the previous round of revisions by the Reviewers 2-4.

Referee 2 discusses in detail novelty of the finding of the present work compared to published comparisons of FN-DMC and CCSD(T), including a paper by some of the present authors, Ref. 9. I generally agree with the overall conclusions that that the authors overemphasize the novelty of their findings. Furthermore, although the history of computational chemistry is full of papers showing disagreements between various methods, and I do not remember any of these papers being published in Nature. The authors made some revisions of the paper, and, with these changes, I mostly agree with what they say, with two exceptions. I still do not agree with the last line of the abstract that states: "Our data contradicts the expectation that the state-of-the-art realizations of the most comprehensive and robust wavefunction methods generally predict identical non-covalent interactions and indicate an unsolved challenge for benchmark approaches." This is not my expectation. Just the opposite, as stated above, I expect that numerical implementations (presumably what the authors mean by "state-of-the-art realizations") of electronic structure methods that agree on smaller systems will agree less well when applied to significantly larger systems. The quoted sentence should simply be removed.

The quoted sentence is removed from the updated manuscript. Perhaps our disagreement with Reviewer 4 in this point lies in the categorization of 'smaller systems' where there is agreement and of 'significantly larger systems' compared to that. The agreement of FN-DMC and CCSD(T) for small systems of few atoms is well-established in the literature. The water-benzene dimer or the systems included in this revision like the benzene dimer include few tens of atoms. Although these systems are significantly larger than few atoms

the agreement is not lost. It is thus important to explore more precisely what is the range of 'significantly larger' especially when these widely trusted approaches become applicable for systems of an additional order of magnitude larger in size, reaching hundred atoms.

Another place that should be modified is the middle paragraph on p. 8, which is constructed in an really convoluted way in order not to say that the paper by some of the present authors, Ref. 9, did report discrepancies between DMC and CCSD(T) for large dimers.

Our aim from the outset has been to achieve the best possible convergence in our work in order to avoid obscuring the (in)consistency of FN-DMC and CCSD(T) with non-converged results. To this end, we note that (i) The mentioned study is a perspective that does not report any new numerical result. (ii) The discrepancies for the S12L set (from literature values) cannot be interpreted in terms of the base methodologies FN-DMC and L-CCSD(T) as both contain substantial uncertainties. (iii) The perspective concludes that we need better converged results from high-level wavefunction methods in particularly moving towards larger molecular complexes, which is what we pursue here.

We have rephrased our description in order to make our point clearer:
"While the average discrepancy of these FN-DMC and local CCSD(T) binding energies was found to be about 2.4 kcal mol^{-1}, it is not possible to make conclusive remarks on the consistency of these results. Uncertainty estimates are unavailable for local CCSD(T), but could be comparable to the average discrepancy, while the error estimates reported for both experimental and FN-DMC energies reach up to a few kcal mol^{-1}".

In the second report, referee 2 still finds that the paper should not be published at all, while referee 3 states that the paper should be submitted to "a high-quality journal with a more specific quantum chemistry focus". Clearly, Nature Communications does not belong to this category. I agree with the recommendation of referee 3, with a minor twist that I would like the authors to modify the statements discussed in the previous paragraph.

In completion of the requested modification we thank Reviewer 4 for joining Reviewer 3 in recommending our manuscript for publication in a high-quality journal covering quantum chemistry.

Reviewer 5 (Remarks to the Author):

The paper describes a discrepancy between two methods claimed to be of benchmark quality, CCSD(T) and FN-DMC, applied to calculations of interaction energies in larger molecular complexes. The authors show that in some of the studied systems, the difference between the results obtained with these two methods is significantly larger than what can be explained by their estimate of the errors of the method (based on calculations in smaller systems), and they suggest that there is some fundamental difference in how these methods describe small and large molecular systems.

It seems the referee assumes our errors are estimated based on smaller systems. Maybe we were not clear in the previous manuscript, as all error estimates were computed system-by-system. Indeed, the error bars grow with system size and complexity from a few tenth of a kcal mol^{-1} at the 20-30 atom range up to 1-2 kcal mol^{-1} for the largest systems. We have now clarified in our revision that the FN-DMC errors account for stochastic uncertainty of the method. We have added the following statement on page 5:

"Meanwhile, the significance of approximations in FN-DMC interaction energies are assessed using statistical measures where error bars indicate 95% confidence intervals."

I would, however, argue that the error estimates on which all the discussion and conclusions stand are wrong, at least at the side of FN-DMC. In fact, there is only very little evidence that FN-DMC and CCSD(T) yield comparable results in small and medium-sized systems, and it is not sufficient for making general, statistically relevant conclusions. The authors cite results obtained in the A24 data set which features only very small systems,(Ref. 42) another work on 6 similar (or identical) molecular complexes (Ref. 2), but no systematic study is available on non-covalent interactions in a large- and diverse-enough set of medium-sized complexes where reliable CCSD(T) results are still available (e.g. at least in the S66 data set).

We thank referee 5 for the suggestion of presenting a more diverse set of molecules. We agree with the referee's suggestion that medium-sized dimers from the S66 would strengthen our message and so we have computed using FN-DMC and local CCSD(T) a subset of S66 molecules which are likely to be the most challenging or controversial due to their size and pi-pi stacking interactions. This includes the parallel displaced benzene, pyridine and uracil dimers (and their mixed combinations) as well as the T-shape configurations of these dimers (which are expected to be simpler).

The new results can be found on pages 9–10 and in Table 1. In summary, using the same methodology as applied to larger systems, we find CCSD(T) and FN-DMC to be statistically indistinguishable for all dimers (*i.e.* within the error bars which are 0.05–0.2 kcal mol^{-1}) with the exception of parallel-displaced benzene where the deviation outside of error bars is only 0.1 kcal mol^{-1} and therefore still considerably small. We discuss this system further in response to the referee's other remarks below. However, it is clear that the premise we present, which is that CCSD(T) and FN-DMC are in general agreement for non-covalent interactions in small-to-medium sized molecules is evidenced by the latest calculations we present as well as the previous cited literature.

Indeed, we would like to bring to the referee's attention that other careful cross-benchmarks exist for carbon dioxide, ammonia, benzene, naphthalene, and anthracene dimers in Ref. [19] in Table S1 of the SI, where agreement is also found, although the structures are less π-stacked. Naphthalene and anthracene are larger molecules than what is present in S66. We have added a footnote on page 4, footnote number 23: "This agreement is in-line with previous predictions of carbon dioxide, ammonia, benzene, anthracene, and

naphthalene dimer interaction energies as shown in the supporting information of Ref. 19."

For systems of this size, the authors showcase the benzene–water complex as an example where there is good agreement between CCSD(T) and FN-DMC. On the other hand, they do omit a much more important and thoroughly studied example, the benzene dimer in the parallel-displaced stacked orientation. It is an important model for pi-pi interactions (which comprise a dominant part of the systems studied in the reviewed paper), and there are very accurate benchmark data available at the CCSD(T) level and beyond. Indeed, it has already been studied also by Monte Carlo methods (Azadi and Cohen 2015, Gasperich and Jordan 2016), and the results are considerably worse there. Specifically, in the former paper, FN-DMC was found to yield an error of almost 1 kcal/mol (with CCSD(T) benchmark of -2.65 kcal/mol) that could be eliminated by applying the backflow transformations that lift the limitations imposed by the fixed-node approximation. The second paper also attributes the error to this approximation. Although a different setup may have been used for the FN-DMC calculations in the present study, it is up to the authors to prove its accuracy.

If the accuracy of FN-DMC (without the backflow transformation correction) is estimated using the available results for benzene dimer as the only model, the error amounts to more than 30%, what would easily explain the observations reported in the paper. It also makes the whole discussion of the differences between CSCD(T) and FN-DMC in the present work worthless. Moreover, it is hard to believe that the omission of the benzene dimer is not intentional. It is so important model system (especially when pi-pi interactions are studied) widely studied in the literature, that it would be a glaring negligence. In the other case, it would be a deliberate manipulation that is even worse.

We did not intentionally neglect the benzene dimer and agree with the referee that it is a better case for π-stacked systems and as such, we have reported it in the revised manuscript using our protocols. Following most recent improvements in the FN-DMC algorithms and by closely monitoring the time-step bias in our calculations, our converged PD benzene dimer interaction energy is -2.38 ± 0.12 kcal mol^{-1} from FN-DMC (0.01 a.u. time-step). The FN-DMC interaction energy is reported for different time-steps and nodal structures in the supplementary material also. We report this and all FN-DMC interaction energies with 95% confidence interval (or 2σ). Considering the error bars on the CCSD(T) result also, there remains only 0.1 kcal mol^{-1} difference between the results. We find this difference to be very small in absolute terms and supports the message in our paper that larger interaction energies are more workable for pinning down the deviation between CCSD(T) and FN-DMC. From the outset, a driving motivation in our work has been to go beyond such 'small' interaction energies because small differences in an interaction energy obscure the limits of numerical precision, theoretical approximations, and calculation settings. We feel we are transparent in this regard and thank the referee for their suggestion which has strengthened the manuscript.

We also acknowledge that the referee points to two studies and in particular the work of Azadi and Cohen (2015). The result that is reported with back-flow transformation is -2.7 ± 0.3 kcal mol^{-1}, where the error

bar is 1σ. This stochastic error is so large as to render the result statistically indistinguishable from either our FN-DMC result or CCSD(T). We are also aware of another result which addresses the fixed node constraint from S. Sorella and co-workers (S. Sorella, M. Casula, and D. Rocca, J. Chem. Phys. 127, 14105 (2007)) and which is also mentioned in the concluding remarks by Gasperich and Jordan (2016). In that work, the result is -2.2 ± 0.3 kcal mol^{-1} but once again, the stochastic error is too large for making conclusive remarks. Note that our error bar at 1σ is 0.06 kcal mol^{-1} which is significantly smaller and is achieved by employing the latest implementations which improve the efficiency of DMC. As a result, we were able to run a simulation $\times 25$ more expensive. Gasperich and Jordan report a result with small enough uncertainty which is -1.97 ± 0.09 kcal mol^{-1} and is slightly lower than ours. However, we do not see fit to discuss that result either in our manuscript for the following reasons: the geometry is different, localized basis sets are used which are possibly not as well-converged as the splines that we use and in addition, their result pre-dates the improvements in DMC algorithm so there could be a bias in the Jastrow optimization and more bias in the time-steps. All of these factors could make up for the difference with respect to our FN-DMC prediction. On the whole, the S66 results we hereby provide should serve as the most reliable FN-DMC references to date for those molecules.

If the authors want to discuss the errors in large systems, they should start with validating the method in medium-sized systems first. Here, a statistically significant number of systems (several tens, including pi-pi complexes such as benzene dimer, stacked nuceobases, etc.) should be computed with exactly the same FN-DMC setup used for the large ones. Until the issues in smaller systems are characterized and resolved, the calculations on large systems are worthless.

We have executed the referee's recommendation and find, as we expected, that CCSD(T) and FN-DMC interaction energies are statistically or thermodynamically (< 0.6 kcal mol^{-1}) consistent across the S66 subset of aromatic pi-stacked and T-shape complexes. The new FN-DMC interaction energies will be a useful reference for medium-sized dimers indeed. However, we strongly believe that the community benefits far more from focusing on larger interaction energies with more tangible deviations. These are ultimately easier to use for understanding and navigating as-yet unchallenged approximations as different communities engage in the puzzle.

On the basis of these findings, I can not recommend the present manuscript for publication neither here, nor in any other journal. It could possibly be pared down to a comparison of results with unknown accuracy on the FN-DMC side if a honest overview of smaller models is included, and such a study would belong to a specialized computational chemistry journal.

We hope we have clarified and demonstrated the precision of both our FN-DMC and local CCSD(T) calculations and the accompanying uncertainty measures. With the computation of medium-sized dimers using FN-DMC, we show that the FN-DMC protocol we apply throughout is reliable and robust. We also

would like to note here and in the revised manuscript that in the time that this work has been under review and revision, two independent works have been published [1, 2] with data in agreement with ours for a subset of the systems studied in our manuscript. Additionally, three more studies used our results as benchmark for the accuracy assessment of lower cost methods [3, 4, 5]. On top of these, four additional papers cite our pre-print highlighting the challenging nature of the presented systems and as a motivation for further advancements in highly-accurate reference methods.[6, 7, 8, 9] This extensive short term activity in the literature demonstrates the real impact of this study.

As it stands, the difference between CCSD(T) and FN-DMC for a few systems found here is a challenge that is worthy of wider attention given the benchmark status and trust that is given to either of these methods both by experimentalists and computational modellers.

References

[1] Anouar Benali, Hyeondeok Shin, and Olle Heinonen. Quantum Monte Carlo benchmarking of large noncovalent complexes in the L7 benchmark set. *J. Chem. Phys.*, 153:194113, 2020.

[2] Francisco Ballesteros, Shelbie Dunivan, and Ka Un Lao. Coupled cluster benchmarks of large noncovalent complexes: The L7 dataset as well as DNA–ellipticine and buckycatcher–fullerene. *J. Chem. Phys.*, 154(15):154104, 2021.

[3] Sebastian Ehlert, Uwe Huniar, Jinliang Ning, James W. Furness, Jianwei Sun, Aaron D. Kaplan, John P. Perdew, and Jan Gerit Brandenburg. r2SCAN-D4: Dispersion corrected meta-generalized gradient approximation for general chemical applications. *J. Chem. Phys.*, 154:061101, 2021.

[4] Stefan Grimme, Andreas Hansen, Sebastian Ehlert, and Jan-Michael Mewes. r2SCAN-3c: A ”Swiss army knife” composite electronic-structure method. *J. Chem. Phys.*, 154:064103, 2021.

[5] Timothy J. Daas, Eduardo Fabiano, Fabio Della Sala, Paola Gori-Giorgi, and Stefan Vuckovic. Noncovalent interactions from models for the Møller–Plesset adiabatic connection. *arXiv e-prints*, page arXiv:2104.04793, 2021.

[6] László Gyevi-Nagy, Mihály Kállay, and Péter R. Nagy. Accurate reduced-cost CCSD(T) energies: parallel implementation, benchmarks, and large-scale applications. *J. Chem. Theory Comput.*, 17:860, 2021.

[7] Adriana Cabrera-Ramírez, Daniel J Arismendi-Arrieta, Alvaro Valdés, and Rita Prosmiti. Exploring CO2@si clathrate hydrates as CO2 storage agents by computational density functional approaches. *ChemPhysChem*, 22:359, 2021.

[8] Jérôme F Gonthier, Maxwell D Radin, Corneliu Buda, Eric J Doskocil, Clena M Abuan, and Jhonathan Romero. Identifying challenges towards practical quantum advantage through resource estimation: the measurement roadblock in the variational quantum eigensolver. *arXiv preprint arXiv:2012.04001*, 2020.

[9] Eno Paenurk and Peter Chen. Modeling gas-phase unimolecular dissociation for bond dissociation energies: Comparison of statistical rate models within rrkm theory. *J. Phys. Chem. A*, 125:1927, 2021.

REVIEWERS' COMMENTS

Reviewer #5 (Remarks to the Author):

The paper had been improved a lot by the inclusion of the medium-sized systems from the S66 data set, and by more comprehensive references to related works.

However, this new data only confirm my concerns about the accuracy of FN-DMC and its misleading interpretation in this paper. The authors now report a difference between CCSD(T) and FN-DMC in parallel-displaced benzene dimer 0.29 kcal/mol (neglecting the error bars). This is, in my opinion, rather poor agreement - many DFT methods now yield error < 0.1 kcal/mol here. This results seriously questions the accuracy of the used FN-DMC setup for pi-pi interactions, and the discrepancies found in the larger systems are likely only an extrapolation of this issue.

The presentation of this result as a small difference (e.g. in Fig. 1) on the basis of the delta_min error is misleading - it would only grow if the calculations had narrower error bars.

In the end, the new data only highlight the bias in the interpretation of the results in the paper, neglecting obvious errors in the smaller systems but 'discovering' serious disagreement in the larger ones.

The same results can be interpreted very differently. For example, the 0.29 kcal/mol difference in PD benzene dimer is 11% of the CCSD(T) interaction energy, and the same relative error in coronene dimer is 12%. The authors describe these two systems very differently, with the first one being "consistent" and the latter "inconsistent", but on the basis of these relative errors, they are both described with the same accuracy and there is thus no surprising difference between small and large systems. Furthermore, because the CCSD(T) result in benzene dimer is more reliable than FN-DMC, the error here can be attributed to this method, and it is thus likely that the discrepancies in the larger systems can be attributed to the error of FN-DMC.

An honest conclusion of the paper would be that the FN-DMC method as used here is not mature enough to describe even rather small systems such as benzene dimer with true benchmark accuracy, and the errors in larger systems are consistent with these findings.

In the light, of the arguments provided above, I can only recommend this paper to be rejected.

Reviewer #6 (Remarks to the Author):

According to the Editor's invitation, I have been brought into this discussion to express my opinion on the disagreement between the Authors of the Paper entitled "Interactions between Large Molecules: Puzzle for Reference Quantum-Mechanical Methods" and some of the referees, in particular, "Referee 5".
As such, I have read both the Article and the "Referee 5" report very carefully, and I will express my opinion on this matter below. However, in general, I will only briefly comment on the suitability of this Article for publication in Nature Communications. Still, I will not specifically take a side on the matter since I have not been specifically asked to do so, and I also believe this decision belongs ultimately to the Editors.

As far as the disagreement that I was called in to judge, the main topic under contention is whether the disagreement between the CCSD(T) and the FN-DMC results for some medium to large-size system that the Authors found and reported as the main conclusion of this Paper bears some significance for computational chemistry research. To this purpose, I believe Referee 5 was indeed on the right track when he initially requested data on small to medium-sized molecules. Specifically, the parallel-displaced benzene dimer, which is notoriously tricky for electronic structure methods, and the S66 database, which is widely considered a reliable database of non-covalent interactions. As their latest revision of the Paper, the Authors presented convincing evidence from these same systems supporting their previous calculations and their main conclusion.

As I mentioned above, I carefully analyzed the Authors' data in the main text and the supporting information, searching for eventual sources of errors that the Authors might have missed. While I certainly tried hard, I could not find any compelling evidence that can justify the differences between the CCSD(T) and FN-DMC results for the three systems highlighted by the Authors as "inconsistent" in their Paper. Coming from a wave function background, I am of the personal opinion that the CCSD(T) results should be of superior quality to FN-DMC, specifically because of the rigid ansatz of the fixed-node approximation. However, it is impossible to explain these discrepancies without a bias, favoring either one of the two methods. In particular, as a counterargument to my general feeling, it is certainly possible that long-range electron correlation and missing higher-order contributions can skew the CCSD(T) results. In this regard, I specifically appreciate and fully support the following sentence in the Article: "Therefore, despite our best efforts to suppress all controllable sources of error, the marked disagreement of FN-DMC and CCSD(T) prevents us from providing conclusive reference interaction energies for these three complexes. Such large differences in interaction energies surpass the widely-sought 1 kcal mol−1 chemical accuracy and indicate that the highest level of caution is required even for our most advanced tools when employed at the hundred-atom scale."

Finally, as a side-note, I find the DFT data a bit puzzling. In the first place, since the Authors used the same exchange–correlation functional (PBE0), this data compares directly the -D4 and -MBD corrections, as also stated in the main text. In general, I would consider both these methods at least one level below the benchmark methods, and it is puzzling that their disagreement is in line with the disagreement among the benchmarks. Is there a physical reason behind it, or is it just a case of a broken clock being correct twice a day? Hard to say with such small information available and certainly an exciting research avenue to explore in the future. In general, however, I would be very worried about drawing any benchmark-level conclusion based on DFT results, even if a more extensive statistical study is to be performed. At this stage, I believe reporting the DFT results here is more to present the oddity rather than to give genuine "insights" as hinted by the Authors.

In summary, I applaud the work of all Referees and all Authors throughout this process. Even without being able to see the first version of the submission, I believe the current version of the Article to be much improved, with essential data that was not presented in the first version. At this stage, I carefully checked both the CCSD(T) and the FN-DMC procedures used by the Authors, and I could not find any inconsistencies. The results and conclusions of the Paper appear to be real effects that cannot be explained at present.

Would this Paper be suitable for publication on Nature Communications, or is it rather more ideal for a more specialized journal? Again, it is not my role to decide. Still, since a couple of referees have also brought up the topic, I would say that I believe the results presented in the Paper and its conclusion are extremely valuable in the field of electronic structure calculations and will certainly sparkle substantial follow-up investigations on similar systems of larger size.

Please find below our point by point reply to the Referees' comments made to our provisionally accepted manuscript.

===== Referees' comments =====

Reviewer 5 (Remarks to the Author):

The paper had been improved a lot by the inclusion of the medium-sized systems from the S66 data set, and by more comprehensive references to related works.

However, this new data only confirm my concerns about the accuracy of FN-DMC and its misleading interpretation in this paper. The authors now report a difference between CCSD(T) and FN-DMC in parallel-displaced benzene dimer 0.29 kcal/mol (neglecting the error bars). This is, in my opinion, rather poor agreement - many DFT methods now yield error < 0.1 kcal/mol here. This results seriously questions the accuracy of the used FN-DMC setup for pi-pi interactions, and the discrepancies found in the larger systems are likely only an extrapolation of this issue.

We are thankful for the advice on including medium-sized dimers and agree that it has added a lot of value to the manuscript. The interpretation of referee 5 here is unsubstantiated in our opinion. Our view is strongly supported by referee 6 who agrees that our data in medium-sized dimers supports and strengthens our main conclusion.

The presentation of this result as a small difference (e.g. in Fig. 1) on the basis of the deltamin error is misleading - it would only grow if the calculations had narrower error bars.

DMC and CCSD(T) have known sources of uncertainty, which we have carefully estimated. Thus, Δ_{min} accounts for any disagreement between the two methods which exceeds the controllable uncertainties. A follow-up work has already picked up our measure of errors [1]. As Δ_{min} is defined and used throughout our work, we feel it is suitable in this figure also.

In the end, the new data only highlight the bias in the interpretation of the results in the paper, neglecting obvious errors in the smaller systems but 'discovering' serious disagreement in the larger ones.

The same results can be interpreted very differently. For example, the 0.29 kcal/mol difference in PD benzene dimer is 11% of the CCSD(T) interaction energy, and the same relative error in coronene dimer is 12%. The authors describe these two systems very differently, with the first one being "consistent" and the latter "inconsistent", but on the basis of these relative errors, they are both described with the same accuracy and there is thus no surprising difference between small and large systems. Furthermore, because the CCSD(T) result in benzene dimer is more reliable than FN-DMC, the error here can be attributed to this method, and it is thus likely that the discrepancies in the larger systems can be attributed to the error

of FN-DMC.

This interpretation is built on the unsupported assumption that CCSD(T) is better in small systems, and it ignores the intrinsic uncertainties that both DMC and CCSD(T) have and that we have carefully evaluated in our paper.

An honest conclusion of the paper would be that the FN-DMC method as used here is not mature enough to describe even rather small systems such as benzene dimer with true benchmark accuracy, and the errors in larger systems are consistent with these findings.

In the light, of the arguments provided above, I can only recommend this paper to be rejected.

We have done our best to carefully consider the opinion of referee 5 but it seems that ultimately we understand FN-DMC differently. We sincerely thank the referee for his/her time in engaging with our work.

Reviewer 6 (Remarks to the Author):

According to the Editor's invitation, I have been brought into this discussion to express my opinion on the disagreement between the Authors of the Paper entitled "Interactions between Large Molecules: Puzzle for Reference Quantum-Mechanical Methods" and some of the referees, in particular, "Referee 5". As such, I have read both the Article and the "Referee 5" report very carefully, and I will express my opinion on this matter below. However, in general, I will only briefly comment on the suitability of this Article for publication in Nature Communications. Still, I will not specifically take a side on the matter since I have not been specifically asked to do so, and I also believe this decision belongs ultimately to the Editors.

As far as the disagreement that I was called in to judge, the main topic under contention is whether the disagreement between the CCSD(T) and the FN-DMC results for some medium to large-size system that the Authors found and reported as the main conclusion of this Paper bears some significance for computational chemistry research. To this purpose, I believe Referee 5 was indeed on the right track when he initially requested data on small to medium-sized molecules. Specifically, the parallel-displaced benzene dimer, which is notoriously tricky for electronic structure methods, and the S66 database, which is widely considered a reliable database of non-covalent interactions. As their latest revision of the Paper, the Authors presented convincing evidence from these same systems supporting their previous calculations and their main conclusion.

We are delighted that referee 6 is convinced by the results and agrees that our main conclusion is well-evidenced.

As I mentioned above, I carefully analyzed the Authors' data in the main text and the supporting information, searching for eventual sources of errors that the Authors might have missed. While I certainly tried

hard, I could not find any compelling evidence that can justify the differences between the CCSD(T) and FN-DMC results for the three systems highlighted by the Authors as "inconsistent" in their Paper. Coming from a wave function background, I am of the personal opinion that the CCSD(T) results should be of superior quality to FN-DMC, specifically because of the rigid ansatz of the fixed-node approximation. However, it is impossible to explain these discrepancies without a bias, favoring either one of the two methods. In particular, as a counterargument to my general feeling, it is certainly possible that long-range electron correlation and missing higher-order contributions can skew the CCSD(T) results. In this regard, I specifically appreciate and fully support the following sentence in the Article: "Therefore, despite our best efforts to suppress all controllable sources of error, the marked disagreement of FN-DMC and CCSD(T) prevents us from providing conclusive reference interaction energies for these three complexes. Such large differences in interaction energies surpass the widely-sought 1 kcal mol1 chemical accuracy and indicate that the highest level of caution is required even for our most advanced tools when employed at the hundred-atom scale."

We are very thankful for the time and effort referee 6 has invested in comprehensively checking the work. We particularly appreciate the referee's honest acknowledgement that we are each perhaps influenced by the bias of our experience. Indeed, this work was enabled through the close collaboration between authors experienced on either side, in CCSD(T) and FN-DMC. In this way we did our best to strike a fair balance and to be open to criticism of either method.

Finally, as a side-note, I find the DFT data a bit puzzling. In the first place, since the Authors used the same exchange–correlation functional (PBE0), this data compares directly the -D4 and -MBD corrections, as also stated in the main text. In general, I would consider both these methods at least one level below the benchmark methods, and it is puzzling that their disagreement is in line with the disagreement among the benchmarks. Is there a physical reason behind it, or is it just a case of a broken clock being correct twice a day? Hard to say with such small information available and certainly an exciting research avenue to explore in the future. In general, however, I would be very worried about drawing any benchmark-level conclusion based on DFT results, even if a more extensive statistical study is to be performed. At this stage, I believe reporting the DFT results here is more to present the oddity rather than to give genuine "insights" as hinted by the Authors.

We thank the referee for considering this final aspect of our work, which we actually agree is more an oddity. One should indeed not confuse the DFT results as more accurate and therefore insightful, but it is nonetheless interesting to us that the main deviation between MBD and D4 arises in the two-body contributions. We write "To demonstrate the consequences of inconsistent references, Fig. 5 shows interaction energy discrepancies obtained with DFAs ..." on page 20, lines 469–471, in order to make the point that different functionals could appear accurate simply due to the chosen reference. We have changed the subheading to "Insights from experiments and comparisons with density functional approximations" to better express the intention

of this analysis.

In summary, I applaud the work of all Referees and all Authors throughout this process. Even without being able to see the first version of the submission, I believe the current version of the Article to be much improved, with essential data that was not presented in the first version. At this stage, I carefully checked both the CCSD(T) and the FN-DMC procedures used by the Authors, and I could not find any inconsistencies. The results and conclusions of the Paper appear to be real effects that cannot be explained at present.

Would this Paper be suitable for publication on Nature Communications, or is it rather more ideal for a more specialized journal? Again, it is not my role to decide. Still, since a couple of referees have also brought up the topic, I would say that I believe the results presented in the Paper and its conclusion are extremely valuable in the field of electronic structure calculations and will certainly sparkle substantial follow-up investigations on similar systems of larger size.

We are extremely grateful for the very positive recognition given to this work by referee 6 and their thorough assessment. We share the referee's opinion that this work should motivate further follow-up investigations and we look forward to more light being shed on the accuracy of wavefunction-based methods as larger and more challenging systems are computed.

===== End of comments =====

References

[1] Francisco Ballesteros, Shelbie Dunivan, and Ka Un Lao. Coupled cluster benchmarks of large noncovalent complexes: The L7 dataset as well as DNA–ellipticine and buckycatcher–fullerene. *J. Chem. Phys.*, 154(15):154104, 2021.
